# The Role of Long Non-Coding RNA in Atherosclerosis: Mechanism and Intervention of Traditional Chinese Medicine

**DOI:** 10.3390/ijms27073194

**Published:** 2026-03-31

**Authors:** Dongmei Yang, Jingyue Wei, Wanjun Lin, Lingran Feng, Qinhui Tuo

**Affiliations:** 1 Key Laboratory for Quality Evaluation of Bulk Herbs of Hunan Province, School of Pharmacy, Hunan University of Chinese Medicine, Changsha 410208, China; dongmeiy@hnucm.edu.cn (D.Y.); 202207010140@stu.hnucm.edu.cn (J.W.); 202207010402@stu.hnucm.edu.cn (W.L.); 202307010735@stu.hnucm.edu.cn (L.F.); 2 Basic Research Center of Chinese and Western Medicine on the Prevention and Treatment of Vascular Diseases, Hunan University of Chinese Medicine, Changsha 410208, China; 3 School of Pharmaceutical Sciences, Hunan University of Medicine, Huaihua 418000, China

**Keywords:** long non-coding RNAs, vascular endothelial cells, vascular smooth muscle cells, macrophages, atherosclerosis, traditional Chinese medicine

## Abstract

Atherosclerosis (AS) is a cardiovascular disease characterized by diverse etiological factors and complex pathological mechanisms. In recent years, the role of long non-coding RNAs (lncRNAs) in AS has received increasing attention. Research shows that lncRNAs regulate key biological processes involved in AS, such as vascular endothelial function, proliferation and migration of vascular smooth muscle cells (VSMCs), macrophage polarization, and lipid metabolism, through various mechanisms, including epigenetic modification, transcriptional regulation, and post-transcriptional control. As important components of traditional medicine, Chinese herbal monomers and compounds have been found to modulate the expression of lncRNAs, thereby improving vascular endothelial function, reducing lipid deposition, and inhibiting inflammatory responses, ultimately exerting anti-atherosclerotic effects. This review systematically examines the role of lncRNAs in the disease mechanism of AS and summarizes recent advances in Traditional Chinese Medicine (TCM) interventions targeting lncRNA expression for the treatment of AS, offering new insights and directions for the prevention and management of AS with Chinese medicine.

## 1. Introduction

Atherosclerosis (AS) is a chronic inflammatory disease that affects large and medium-sized arteries. Its main pathological features are abnormal lipid deposition beneath the arterial intima, infiltration of inflammatory cells, and formation of plaque, all of which ultimately lead to narrowing of the vascular lumen and impaired blood flow [1]. AS is the pathological basis of not only cardiovascular disease but also cerebrovascular disease [2]. Atherosclerotic cardiovascular disease (mainly including ischemic heart disease and ischemic stroke) is the main cause of the global burden of disease [3]. According to the latest World Health Organization report, cardiovascular diseases (CVDs) caused approximately 19.8 million deaths globally in 2022, accounting for about 32% of all global mortality. Among these CVD-related deaths, 85% were attributable to heart attacks and strokes [4]. It seriously threatens human health. Numerous studies have shown that the development and progression of AS are closely associated with multiple pathological mechanisms, including disordered lipid metabolism, activation of inflammatory responses, and oxidative stress injury [5,6]. The initial event in AS is endothelial cell dysfunction induced by abnormal hemodynamic shear stress. Damaged vascular endothelial cells (VECs) promote monocyte adhesion. The differentiation of monocytes into macrophages is stimulated by factors such as macrophage colony-stimulating factor and other cytokines. Subsequently, the macrophages engulf oxidized low-density lipoprotein (ox-LDL), undergoing a transformation into foam cells. When these foam cells die, they release cellular contents that are ingested by other macrophages, ultimately leading to the formation of atherosclerotic plaques [7]. These plaques contain vascular smooth muscle cells (VSMCs), collagen, foam cells, and other components [8,9]. Therefore, the initiation and progression of AS involve VECs, macrophages, and VSMCs. Notably, endothelial injury is considered the initiating factor in AS. Macrophages participate throughout the entire pathogenic process, and the inflammatory responses they mediate play a key role in plaque formation and progression. Meanwhile, the migration and proliferation of VSMCs is a key factor in the stability of advanced plaques [10]. A comprehensive understanding of these molecular mechanisms, along with the development of new interventions, is crucial for slowing the progression of AS. Current clinical strategies for managing AS primarily include statin lipid-lowering drugs, antiplatelet therapy, and revascularization procedures such as balloon angioplasty, stent implantation, and bypass grafting [11]. The current treatment methods are widely used, which can not only delay the progression of AS but also significantly reduce the incidence of cardiovascular and cerebrovascular events. However, due to the failure to completely eliminate the risk of morbidity, the related cardiovascular and cerebrovascular diseases are still one of the leading causes of death and disability worldwide. With its unique advantages of a multi-target, multi-pathway action and low side effects, Traditional Chinese Medicine (TCM) has been increasingly applied in the prevention and treatment of AS [12]. Studies have confirmed that various TCM active components and formulas exert anti-AS effects by regulating cellular functions. For instance, icariin alleviates vascular endothelial cell injury [13] and inhibits the abnormal proliferation and migration of VSMCs [14]; salvianolic acid A and danshensu produce protective effects by inhibiting vascular endothelial inflammation [15] and apoptosis [16], respectively; curcumin regulates endothelial cell apoptosis and proliferation, mitigating inflammatory responses [17]; and the compound formula Tongmai Zhuke Decoction inhibits macrophage-mediated inflammation by upregulating lncRNA-Cox2 [18].

It is worth noting that these TCM components often exert anti-AS effects through regulating long non-coding RNAs (lncRNAs)—RNA molecules longer than 200 nucleotides that lack protein-coding capacity but exhibit high stability [19]. Recent studies have indicated that lncRNAs are more abundant than protein-coding genes in the genome and play a key regulatory role in many biological processes, such as differentiation, organ development, cell proliferation, apoptosis, and tissue homeostasis [20]. Consequently, dysregulation of lncRNA expression is closely associated with the initiation, progression, and metastasis of various diseases [21]. Although lncRNAs lack protein-coding capacity, they exert key regulatory roles in various diseases, including AS. A variety of mechanisms have been identified through which this process may occur, including epigenetic modification, transcriptional interference, and post-transcriptional regulation by sequestering microRNAs (miRNAs) or by directly binding to mRNAs [22,23]. Accumulating evidence indicates that lncRNAs orchestrate AS progression by influencing the homeostasis and lipid metabolism of the vasculature’s core cell types, such as VECs, VSMCs, and macrophages [24]. Therefore, lncRNAs have been demonstrated to be promising therapeutic targets for AS treatment [25,26,27]. Previous studies further suggest that TCM can effectively prevent and mitigate AS by regulating the expression and function of lncRNA. This review presents the pivotal roles of lncRNAs in AS pathogenesis and their relevance in TCM-based AS interventions, aiming to clarify the molecular mechanisms through which TCM modulates lncRNAs in AS and to expand potential strategies to prevent and clinically treat this disease.

## 2. LncRNAs and VECs

**Figure 1:**
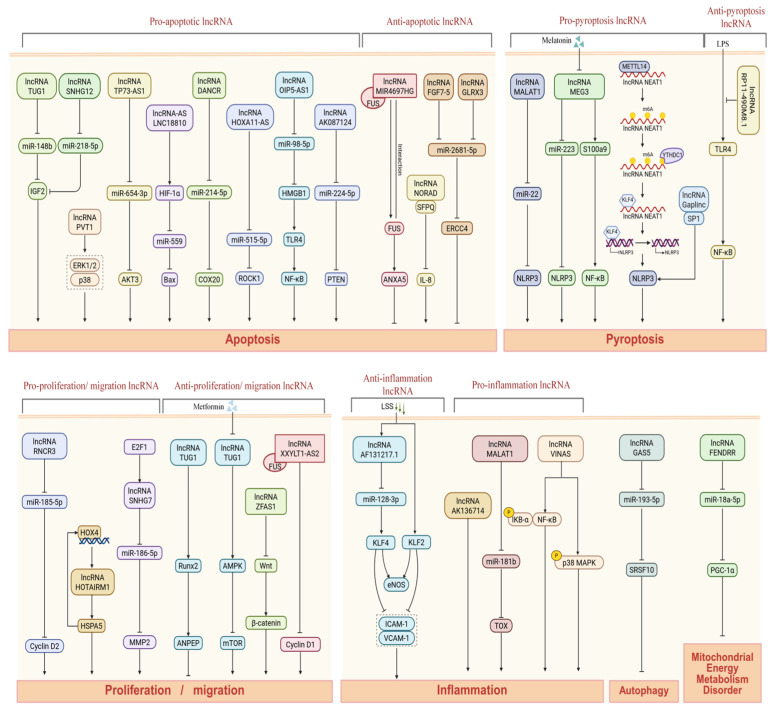
Molecular mechanism of lncRNA regulating VEC function. This schematic illustrates the molecular mechanism of lncRNA in regulating key biological processes (including apoptosis, pyroptosis, proliferation and migration, and inflammatory response) in VECs. In the figure, solid arrows indicate activation or promotion; T-shaped arrows represent inhibition; phosphorylation events are indicated by a circled “P” on arrows; dashed boxes denote key affected indicators; double wavy lines represent double-stranded DNA, and a single wavy line represents modified lncRNA; different color boxes are used to distinguish various biomolecules (such as lncRNA, miRNA, protein, or drug), and arrows indicate the activation, inhibition, or regulation relationship between molecules. In terms of mechanism, most lncRNAs adsorb and inhibit the function of specific miRNAs, thereby removing the inhibition of miRNA on its downstream target gene expression. In addition, there are also epigenetic modifications (m6A modification) and external factors (laminar shear stress (LSS), metformin, and melatonin) that can regulate the above lncRNA-mediated regulatory network, providing potential targets for therapeutic interventions for related diseases. Created in BioRender. Jingyue_Wei. (2026) https://app.biorender.com/illustrations/69be937b21b78d3f82d63c8f (accessed on 10 February 2026).

VECs play a central role in maintaining cardiovascular homeostasis, with their functions encompassing vascular tone regulation, coagulation balance, and inflammatory control. In the pathological process of AS, endothelial dysfunction serves as a key initiating event [28]. Notably, several lncRNAs are specifically enriched in endothelial cells and have been shown to modulate critical processes, including endothelial senescence, inflammation, apoptosis, pyroptosis, proliferation, migration, and barrier function. Excessive apoptosis and pyroptosis can compromise endothelial integrity, trigger vascular inflammation, and exacerbate endothelial dysfunction, thereby promoting lipid deposition, plaque formation, and the progression of AS. Collectively, these findings position lncRNAs as critical molecular hubs linking endothelial dysfunction to the pathogenesis of AS (Figure 1, Table 1).

### 2.1. LncRNAs and VECs Apoptosis

VEC apoptosis is a crucial initiating event in the early phase of AS, which impairs endothelial barrier integrity and creates a pathological microenvironment conducive to subsequent lipid deposition and inflammatory cell infiltration in the vascular wall [60]. Accumulating evidence has clarified that lncRNAs play a pivotal regulatory role in ox-LDL-induced VEC apoptosis in in vitro experimental models, mainly through acting as competitive endogenous RNAs (ceRNAs) or directly modulating classic inflammatory and stress signaling pathways.

In terms of pro-apoptotic regulation, lncRNAs primarily function through two major mechanisms. First, certain lncRNAs induce apoptosis by activating key inflammation and stress signaling pathways. For instance, LncRNA PVT1 is directly involved in ox-LDL-induced HUVEC apoptosis in vitro by promoting the activation of ERK1/2 and p38 signaling pathways in cultured cells, and this pro-apoptotic effect in vitro provides a potential molecular link to AS progression, but its in vivo relevance to plaque development requires verification through animal experiments [29]. Secondly, a more common pro-apoptotic mechanism involves the classic ceRNA “sponge” mechanism. In this process, lncRNAs competitively bind to microRNAs (miRNAs), thereby relieving the inhibition of these molecules on downstream pro-apoptotic target genes, thereby indirectly inducing apoptosis [61]. Specifically, lncRNA TP73-AS1 adsorbs miR-654-3p in human aortic endothelial cells (HAECs) to positively regulate the expression of its downstream target AKT3, thus promoting ox-LDL-induced apoptosis of cultured HAECs in vitro [30]; lncRNA-ASLNC18810 in apoptosis-resistant endothelial cells (ARECs) indirectly positively regulates the expression of the pro-apoptotic protein Bax through the HIF-1α/miR-559/Bax axis, leading to increased apoptosis of cultured VECs [31]. In addition, lncRNA DANCR in HUVECs and VSMCs targets the miR-214-5p/COX20 axis [32]. lncRNA HOXA11-AS directly inhibits miR-515-5p in HUVECs to upregulate ROCK1 expression and downregulate eNOS expression [33], and lncRNA SNHG12 in HUVECs acts on the miR-218-5p/IGF2 axis [34]. At the same time, LncRNA-TUG1 exerts an apoptotic effect on ox-LDL-induced HUVECs in vitro through the miR-148b/IGF2 ceRNA axis [35]; lncRNA AK087124 adsorbs miR-224-5p in mouse aortic endothelial cells (MAECs) to regulate the expression of PTEN, thereby modulating the AKT signaling pathway and regulating apoptosis and inflammation of ox-LDL-induced cultured endothelial cells [36]. Notably, lncRNA OIP5-AS1 shows functional complexity in the pro-apoptotic regulation. It downregulates miR-98-5p through the TLR4/NF-κB signaling pathway in vitro, further mediating the expression of HMGB1 and ultimately promoting apoptosis of ox-LDL-induced HUVECs [37].

In contrast to pro-apoptotic effects, another subset of lncRNAs exerts powerful anti-apoptotic protective effects by inhibiting inflammatory pathways or functioning as ceRNAs. LncRNA NORAD has been confirmed to reduce the senescence and apoptosis of HUVECs by regulating the NF-κB and p53-p21 pathways and may reduce IL-8 by directly interacting with SFPQ protein [38]. LncRNA FGF7-5 and lncRNA GLRX3 jointly target the miR-2681-5p/ERCC4 axis in HUVECs to reduce the apoptosis of cultured HUVECs in vitro [39]; lncRNA MIR4697HG upregulates FUS and downregulates ANXA5 in HUVECs, which protects cultured HUVECs from ox-LDL-induced damage in vitro and alleviates apoptosis and oxidative stress of endothelial cells [40].

In summary, lncRNAs constitute a delicate regulatory system in the process of vascular endothelial cell apoptosis by modulating inflammatory signaling pathways (such as p38 MAPK and NF-κB) and complex ceRNA networks. These molecules can serve as both driving factors of AS and endogenous protective factors. The diversity and complexity of their functions underscore their central position in the pathophysiology of AS. It is worth noting that current research on the regulatory mechanisms of lncRNAs in endothelial cell apoptosis is primarily focused on in vitro cell models. Although these studies have laid a molecular foundation for understanding the early endothelial injury mechanisms of AS, further in vivo animal experiments and clinical cohort studies are needed to elucidate the causal relationships and functional specificity underlying how these in vitro mechanisms translate into the regulation of AS plaque development and progression in vivo.

### 2.2. LncRNAs and VECs Pyroptosis

Inflammasome activation (for instance, by NLRP3) triggers pyroptosis, a form of inflammatory cell death mediated by Gasdermin D (GSDMD). Its characteristics include cell membrane pore formation, cell swelling, and rapid lysis, accompanied by the release of numerous inflammatory factors [62]. In AS, pyroptosis of VECs directly compromises vascular barrier integrity and promotes the recruitment and infiltration of inflammatory cells (such as monocytes) into plaques, thereby exacerbating the local inflammatory response and inducing plaque instability or even rupture. LncRNAs are pivotal regulators of the pyroptosis pathway, and their imbalanced expression is closely associated with the pathological progression of AS.

Multiple studies have revealed that different lncRNAs regulate endothelial cell pyroptosis through specific molecular mechanisms, with research models including high glucose-induced endothelial injury, general inflammatory injury, and in vivo AS models. First, in high glucose-induced HUVEC injury models, lncRNA MALAT1 was found to positively regulate pyroptosis by competitively binding to microRNA-22 (miR-22) and relieving the inhibitory effect of miR-22 on NLRP3 [41]. Secondly, lncRNA NEAT1, which is upregulated in AS lesions and in vitro ox-LDL-induced endothelial injury models, promotes the assembly of the NLRP3 inflammasome by binding to the transcription factor KLF4, thereby triggering endothelial cell pyroptosis [42]. Additionally, in a high-fat diet-fed mouse AS model, silencing lncRNA Gaplinc can inhibit its interaction with the transcription factor SP1, thereby downregulating the expression of NLRP3, and ultimately reducing endothelial cell pyroptosis and AS plaque development, providing direct in vivo evidence for Gaplinc’s involvement in AS-related pyroptosis [43].

Beyond these direct regulatory mechanisms, studies have also identified lncRNA involvement in indirect or drug intervention regulation of pyroptosis, with partial evidence from AS-related models. For example, melatonin, which has significant anti-inflammatory properties, was found to reduce AS plaques in ApoE^−/−^ mouse models and downregulate the expression of endothelial cell pyroptosis-related genes (NLRP3, GSDMD, and IL-1β), with part of its mechanism of action potentially achieved by interfering with lncRNA MEG3 [44]. Furthermore, in human aortic endothelial cells (HAECs) treated with ox-LDL, MEG3 can bind to and inhibit the function of miR-223 through sequence complementation, thereby upregulating the expression of NLRP3 and promoting pyroptosis, and melatonin has been demonstrated to reverse this process, which indicates that melatonin has the potential to serve as a novel therapeutic strategy for AS by regulating lncRNA-mediated pyroptosis [45]. In addition, the study confirmed that lncRNA RP11-490M8.1 is significantly downregulated in atherosclerotic plaques and serum, and can inhibit lipopolysaccharide-induced pyroptosis of human umbilical vein endothelial cells through the TLR4/NF-κB pathway, which is expected to be a therapeutic target for AS [46].

It should be noted that some of the current evidence on lncRNA regulating endothelial cell pyroptosis comes from non-AS-specific models (e.g., high glucose-induced injury), and the translation of these mechanisms to the in vivo AS microenvironment requires further verification using standardized AS models (such as ApoE^−/−^ or LDLR^−/−^ mice fed a high-fat diet). Future studies should focus on constructing more physiological AS models to clarify the specific regulatory roles of lncRNAs in pyroptosis during AS progression, thereby providing more reliable theoretical support for clinical intervention strategies.

### 2.3. LncRNAs and VECs Proliferation and Migration

Abnormal proliferation and migration of VECs are key pathological processes that drive vascular remodeling and plaque progression in AS. When the endothelial repair mechanism is imbalanced, excessive proliferation leads to intimal thickening, while abnormal migration compromises endothelial barrier integrity, collectively promoting inflammatory infiltration and lipid deposition [63]. As important regulators of this process, lncRNAs bidirectionally regulate the proliferation and migration of VECs through complex molecular networks, and their dysregulation is closely associated with AS progression.

On the one hand, multiple lncRNAs have been reported to regulate VEC proliferation and migration through specific molecular mechanisms, with research models including AS-related models (e.g., ox-LDL-induced endothelial cells and high-fat diet-fed ApoE^−/−^ mice) and non-AS models (e.g., general inflammatory stimulation and gene modification in normal endothelial cells). For example, LncRNA HOTAIRM1 is transcriptionally activated by HOXA4 and forms a positive feedback loop with HOXA4; by upregulating the expression of HSPA5, it promotes the proliferation of HUVECs [47]. Additionally, overexpression of lncRNA RNCR3 in HUVECs drives cell cycle progression through the miR-185-5p/cyclin D2 axis, promoting VEC proliferation and the secretion of pro-inflammatory cytokines such as IL-6, IL-1β, and TNF-α [48]; similarly, lncRNA SNHG7, driven by the transcription factor E2F1 (which is upregulated in AS), upregulates the expression of matrix metalloproteinase MMP2 by adsorbing miR-186-5p in ox-LDL-treated HUVECs, forming the E2F1/SNHG7/miR-186-5p/MMP2 axis that regulates VEC proliferation and migration [49].

On the other hand, another category of lncRNAs with protective functions modulates AS by inhibiting abnormal VEC proliferation and migration. LncRNA ZFAS1 is downregulated in IL-8-stimulated HUVECs, and its reduced expression promotes HUVEC proliferation and adhesion to monocytes by affecting the miR-150-5p/Notch3 signaling axis [50]; besides its involvement in inflammation regulation, lncRNA TUG1, which is highly expressed in AS lesions, has been demonstrated in high-fat diet-induced ApoE^−/−^ mice and ox-LDL-treated HUVECs that its knockdown inhibits HUVEC proliferation and migration. This process involves AMPK/mTOR pathway activation and subsequent autophagy induction, and the anti-AS effect of metformin is partially achieved by targeting TUG1, providing direct evidence for its role in AS. Furthermore, it is found that TUG1 is upregulated in ox-LDL-treated endothelial cells [51]. Silencing TUG1 enhances the proliferation and migration of injured endothelial cells by inhibiting the binding of Runx2 to the ANPEP promoter and downregulating ANPEP expression, suggesting that silencing TUG1 can promote atherosclerotic vascular injury repair and suppress AS progression through the Runx2/ANPEP signaling axis [52]. In ox-LDL-treated HUVECs, overexpression of lncRNA XXYLT1-AS2 not only reduces VCAM-1 and MCP-1 levels but also regulates the VEC cell cycle by directly interacting with the FUS/cyclin D1 complex, playing a protective role at multiple levels, including proliferation, migration, and inflammation, by blocking NF-κB activity [53]. It should be emphasized that some of the current research on lncRNAs regulating VEC proliferation and migration is based on non-AS-specific models, and the translation of these mechanisms to AS pathological processes requires careful validation.

### 2.4. Other Regulatory Mechanisms in VECs

Research has revealed that beyond influencing VEC apoptosis, pyroptosis, proliferation, and migration, lncRNAs also play central roles in the pathogenesis of AS by regulating multiple key biological processes in VECs, including inflammatory responses, autophagy, and energy metabolism.

For example, lncRNA AF131217.1, which is regulated by laminar shear stress (a key physiological factor in AS pathogenesis), inhibits the expression of ICAM-1 and VCAM-1 through the AF131217.1/miR-128-3p/KLF4 axis in ox-LDL-treated HUVECs, maintaining endothelial homeostasis while exerting anti-inflammatory effects [54]. lncRNA AK136714 has been shown to damage the endothelial barrier and aggravate the inflammatory response, thereby promoting AS formation in the ApoE^−/−^ mouse model, and silencing this molecule can effectively reduce endothelial damage and inhibit disease progression [55]. More in-depth mechanistic studies revealed that lncRNA MALAT1 is upregulated in AS and has been observed to participate in endothelial cell inflammation and oxidative stress, which have been induced by oxidized low-density lipoprotein by adsorbing miR-181b and releasing its inhibition on the target gene TOX [56]. The lncRNA VINAS was identified as a key upstream regulator of both the NF-κB and p38 MAPK pathways, and its pro-inflammatory effect significantly aggravated AS; most importantly, in vivo intervention using its specific antagonist LNAgapmeR effectively reduced vascular wall inflammation levels, demonstrating its considerable potential as a therapeutic target [57].

Furthermore, by impairing endothelial cell autophagy via the miR-193-5P/SRSF10 axis, lncRNA GAS5 disrupts autophagic flux, which in turn accelerates the development of AS [58]. Additionally, maintaining endothelial cell energy homeostasis represents another important function of lncRNAs. By acting on the miR-18a-5p/PGC-1α pathway, lncRNA FENDRR effectively reverses mitochondrial energy metabolism disorders induced by ox-LDL, thus exerting an endothelial protective effect [59]. These insights not only elucidate AS pathogenesis but also offer a rationale for novel interventions targeting lncRNAs, holding promise for future treatment strategies.

## 3. LncRNAs and VSMCs

**Figure 2:**
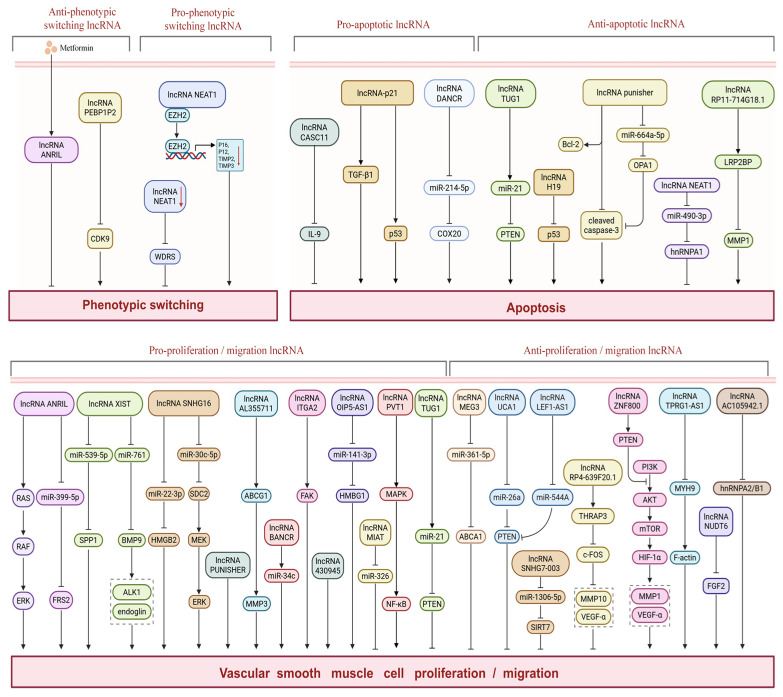
LncRNAs and Vascular Smooth Muscle Cells. This diagram illustrates the molecular mechanism of lncRNA in regulating key biological processes (including phenotypic transformation, osteogenic transformation, apoptosis, proliferation, and migration) in VSMCs. In the figure, solid arrows indicate activation or promotion; T-shaped arrows represent inhibition; phosphorylation events are indicated by a circled “P” on arrows; dashed boxes denote key affected indicators; double wavy lines represent double-stranded DNA. In phenotypic switching, the regulatory role of lncRNAs was delineated into anti-phenotypic switching (e.g., ANRIL and PEBP1P2) and pro-phenotypic switching (e.g., NEAT1) categories. In the apoptosis pathway, the downstream effector molecules of pro-apoptotic (e.g., CASC11, p21, etc.) and anti-apoptotic (e.g., H19 and Punisher) lncRNAs were identified. In the proliferation and migration module, the signal cascades of lncRNAs that promote proliferation and migration (e.g., ANRIL and XIST) and anti-proliferation and migration (e.g., MEG3 and UCA1) were divided, covering classic pathways such as RAS/RAF/ERK and PI3K/AKT/mTOR.Created in BioRender. Jingyue_Wei. (2026) https://app.biorender.com/illustrations/69becf1fe28763a6dd716a10 (accessed on 10 February 2026).

VSMCs are the major cellular component of the vascular media, maintaining vascular tone via a contractile phenotype under physiological conditions [64]. In AS, VSMCs undergo phenotypic transformation to a synthetic type upon stimulation by PDGF, Ang II, and other factors, gaining the capacities of proliferation, migration, and extracellular matrix synthesis—an initiating step of AS pathogenesis [65]. Synthetic VSMCs migrate from the media to the intima and undergo abnormal proliferation, directly causing intimal thickening and luminal stenosis; their secreted collagen and other components form the plaque fibrous cap (initially stabilizing plaques but exacerbating vascular remodeling and lipid core expansion when over-secreted). Additionally, oxidative stress and inflammatory factors induce VSMCs apoptosis imbalance: uncleared apoptotic cells trigger secondary necrosis, aggravate the inflammatory microenvironment, and elevate plaque instability, while matrix metalloproteinases (MMPs) released by apoptotic VSMCs degrade the extracellular matrix and destroy fibrous cap structural integrity [66]. Mounting studies have confirmed that lncRNAs exert critical regulatory effects on VSMCs phenotypic transformation, proliferation, migration, and apoptosis through complex molecular networks, profoundly modulating the entire AS process and emerging as potential therapeutic intervention targets (Figure 2, Table 2).

### 3.1. LncRNAs and VSMCs Phenotypic Transformation

Phenotypic switching of VSMCs from a contractile to a synthetic state in response to vascular injury or growth factor stimulation is a core adaptive characteristic to microenvironmental changes in AS [100]. This transformation endows VSMCs with proliferative and migratory abilities, leading to intimal hyperplasia and vascular remodeling, which are key drivers of atherosclerotic plaque formation [101]. LncRNAs are now recognized as central regulators of this process, and their underlying molecular mechanisms of regulating VSMCs phenotypic transformation have become a key research focus in the AS field.

Among these, lncRNA ANRIL has been confirmed to inhibit AS progression. Studies based on ApoE^−/−^ mouse AS models and in vitro VSMCs models of AS have found that after metformin intervention, ANRIL expression levels are significantly increased, effectively inhibiting the switching of VSMCs to a synthetic phenotype, and ultimately preventing further development of atherosclerotic plaques [67]. Additionally, lncRNA PEBP1P2 was found to effectively inhibit VSMCs phenotypic conversion, abnormal proliferation, and migration by targeting the cyclin-dependent kinase 9 (CDK9) signaling pathway [68]. The research is based on AS-related experimental models. Notably, lncRNA NEAT1 exhibits dual mechanisms in regulating VSMCs phenotype. When specific knockout of NEAT1 in mice significantly improves the degree of intimal hyperplasia following vascular injury, and simultaneously, if NEAT1 is lowly expressed, WDR5 is released and activates the promoter region of VSMCs contraction-related genes, promoting VSMCs maintenance of the contractile phenotype [69]. However, Yin et al.’s research based on AS VSMCs models revealed another aspect of NEAT1, as it recruits EZH2 through scaffolding, and subsequently enhances VSMCs proliferation, migration, and osteogenic differentiation capabilities through epigenetic mechanisms, which provides a new explanation for NEAT1’s complex role in AS [70].

The collective evidence from these studies, all based on direct AS models (in vivo AS animal models/in vitro AS-related VSMCs models), suggests a role for lncRNAs in various levels of VSMCs phenotypic transformation through complex regulatory networks, involving processes such as proliferation, migration, and osteogenic differentiation. This forms a solid basis for elucidating the pathogenesis of AS and formulating novel treatment approaches.

### 3.2. LncRNAs and VSMCs Proliferation and Migration

Abnormal VSMCs proliferation and migration are pivotal links in AS initiation and progression, closely associated with VSMCs phenotypic switching to a synthetic type. Pathologically activated VSMCs migrate to the intima and proliferate abnormally, participating in fibrous cap formation, while excessive proliferation directly induces intimal thickening and lumen stenosis. Moreover, synthetic VSMCs secrete extracellular matrix (ECM) and MMPs, which not only mediate plaque remodeling but also disrupt plaque stability, ultimately promoting AS plaque progression, rupture, and thrombosis. LncRNAs precisely regulate VSMCs proliferation and migration through intricate regulatory networks, and their regulatory effects run through the entire process of AS plaque formation, progression, and rupture.

Multiple studies have confirmed that a series of lncRNAs positively regulate VSMCs proliferation and migration by acting as ceRNAs, activating key signaling pathways, or directly interacting with functional proteins, thereby accelerating AS progression. Among these, the ceRNA mechanism represents an important functional pathway for lncRNAs. Unless otherwise specified, the following findings have been validated in AS-specific experimental models (e.g., ApoE^−^/^−^ mouse AS models and ox-LDL-induced VSMCs models). For example, LncRNA ANRIL adsorbs miR-399-5p through a sponge mechanism and relieves its inhibition on the RAS/RAF/ERK signaling pathway, thereby promoting the proliferation and migration of human aortic VSMCs (HA-VSMCs) induced by ox-LDL [71]. By sequestering miR-539-5p, LncRNA XIST functions as a ceRNA, which ultimately promotes VSMCs proliferation and migration under ox-LDL stimulation via the miR-539-5p/SPP1 axis [72]. Additionally, its knockout can inhibit the BMP9/ALK1/endoglin pathway by promoting miR-761 expression, thereby inhibiting HA-VSMCs proliferation and migration [73]. As a ceRNA for miR-22-3p, the function of lincRNA SNHG16 is to accelerate AS plaque formation and enhance ox-LDL-activated VSMCs proliferation and migration by regulating HMGB2 expression [74]. Furthermore, downregulation of SNHG16 has been demonstrated to inhibit the potentiation of the MEK/ERK axis through the targeting of the miR-30c-5p/SDC2 axis, thereby preventing ox-LDL-induced proliferation and migration of VSMCs in cerebral AS models [75]. LncRNA MIAT promotes AS progression by competitively adsorbing miR-326, and miR-326 upregulation can reverse MIAT-mediated promotion of VSMCs migration [76].

Beyond the ceRNA mechanism, some lncRNAs function by directly or indirectly regulating signaling pathways. For example, the confirmed functions of LncRNA PUNISHER include promoting cellular proliferation and migration, as well as activating mitochondrial fission and apoptosis-related pathways [77]. LncRNA AL355711 promotes VSMCs migration through the ABCG1/MMP3 pathway [78]. Additionally, overexpression of lncRNA BANCR can induce human aortic smooth muscle cells (HASMCs) proliferation by downregulating miR-34c methylation, and reverse miR-34c effects on HMGB1, TNF-α, and Bcl-2 expression [79]. LncRNA-ITGA2 directly binds to its complementary sequence in the nucleus to increase ITGA2 transcriptional activity; the two synergistically activate the ITGA2-FAK signaling pathway and promote HA-VSMCs proliferation and migration [80]. Furthermore, it is evident that lncRNA OIP5-AS1 exerts an indirect influence on HMGB1 expression through the targeting of miR-141-3p, a process which has been demonstrated to promote proliferation and migration of VSMCs, whilst concomitantly inhibiting apoptosis [81]. LncRNA 430945 expression is upregulated in AS, and its downregulation can significantly inhibit Ang II-induced VSMCs proliferation and migration in vascular injury models (Ang II-induced and AS risk factor) [82]. Silencing lncRNA PVT1 impedes HA-VSMCs proliferation while facilitating apoptosis through down-modulation of the MAPK/NF-κB axis [83]. Some researchers have found that lncRNA TUG1, as a ceRNA, competes with PTEN to bind miR-21, promoting VSMCs proliferation while inducing their apoptosis, and participating in the AS process in ApoE^−^/^−^ mouse AS models and ox-LDL-induced HA-VSMCs models [84].

Contrasting with AS-promoting lncRNAs, another category of lncRNAs inhibits abnormal VSMCs proliferation and migration through similar but opposite molecular mechanisms, exerting protective effects against AS. LncRNA MEG3, as a ceRNA for miR-361-5p, participates in regulating VSMCs proliferation by affecting ABCA1 expression, and its inhibition can aggravate ox-LDL-induced VSMCs proliferation [85]. It has been demonstrated that lincRNA UCA1 functions as an endogenous sponge for miR-26a, thereby modulating its expression levels; this regulatory mechanism effectively counterbalances the inhibitory effect of miR-26a on the PTEN target gene, thus contributing to the suppression of aberrant VSMCs proliferation [86]. LncRNA LEF1-AS1 constrains VSMCs proliferation and motility via activation of the PTEN axis; this restraint is annulled by miR-544a overexpression [87]. Through the miR-1306-5p/SIRT7 signaling axis, lncRNA SNHG7-003 suppresses the proliferation, migration, and invasion of VSMCs [88]. LncRNAs TPRG1-AS1 and ENST00000444488.1 both physically interact with MYH9. Among them, TPRG1-AS1 facilitates MYH9 proteolysis, thereby impairing F-actin stress-fiber assembly and suppressing neointimal hyperplasia in vivo [89]. Likewise, lncRNA RP4-639F20.1 constrains VSMCs proliferation and motility by engaging THRAP3, modulating the c-FOS cascade, and repressing MMP10 and VEGF-α expression [90]. Concurrently, lncRNA ZNF800 overexpression attenuates AKT/mTOR/HIF-1α signaling, downregulates VEGF-α and MMP1 transcripts, and consequently suppresses VSMCs proliferation and migration [91]. As the antisense counterpart of FGF2, lncRNA NUDT6 knockout evokes SMC survival and locomotion in vascular disease progression models (AS-relevant), underscoring its putative therapeutic utility [92]. LncRNA AC105942.1 mitigates Ang-II-elicited VSMCs proliferation via negative regulation of hnRNPA2B1 in vascular injury models (Ang II-induced and AS risk factor) [93].

### 3.3. LncRNAs and VSMCs Apoptosis

VSMCs apoptosis exerts a complex, dual role in AS pathogenesis. Moderate VSMCs apoptosis is beneficial for maintaining vascular wall cellular homeostasis and preventing vascular stenosis caused by excessive VSMCs proliferation [102], whereas excessive apoptosis impairs the structural integrity of the plaque fibrous cap and markedly increases the risk of plaque instability and rupture [103]. Recent research has revealed that lncRNAs finely regulate VSMCs apoptosis through diverse molecular mechanisms, and this precise regulation enables lncRNAs to profoundly influence the pathological progression of AS by modulating the balance of VSMCs apoptosis.

LncRNA DANCR serves as a ceRNA targeting the miR-214-5p/COX20 axis, and its downregulation can reduce apoptosis, increase ox-LDL-treated VSMCs viability, and promote AS progression in ox-LDL-induced AS VSMCs models [32]. LncRNA CASC11 overexpression ameliorates AS-related pathological alterations via IL-9 downregulation, suppression of VSMCs proliferation, and facilitation of apoptosis in ox-LDL-induced AS VSMCs models [94]. Furthermore, lncRNA-p21 potently restrains VSMCs proliferation and provokes apoptosis by potentiating p53 transcriptional activity, thereby modulating vascular neointimal formation in ApoE^−^/^−^ mouse AS models [95]. In addition, the study also found that overexpression of lncRNA-P21 can prevent apoptosis in VSMCs in thoracic aortic aneurysm models (AS comorbidity-related vascular injury models) by suppressing caspase-3 activity and lowering Bax expression while simultaneously elevating Bcl-2 and mTOR levels [96]. Although these pro-apoptotic lncRNAs can temporarily inhibit excessive cell proliferation, they may lead to fibrous cap thinning due to excessive apoptosis induction, ultimately exacerbating plaque instability.

Contrary to the above effects, another category of lncRNAs inhibits VSMCs apoptosis through multiple molecular mechanisms, maintains fibrous cap integrity, and plays protective roles in AS. Studies have demonstrated that lncRNA PUNISHER functions as a ceRNA by directly interacting with miR-664a-5p, thereby modulating the mitochondrial fusion protein OPA1; its overexpression can markedly suppress neointimal hyperplasia and VSMCs apoptosis [77]. In addition, knockdown of lncRNA H19 can alleviate AS development by enhancing p53 activity, inhibiting abnormal VSMCs proliferation, and promoting apoptosis in ApoE^−/−^ mouse AS models [97]. RP11-714G18.1 inhibits HA-VSMCs apoptosis by targeting LRP2BP expression, downregulating MMP1, and participates in AS formation and development in human atherosclerotic plaque-derived VSMCs models [98]. In addition, studies have confirmed that miR-490-3p is an inhibitory target of NEAT1; overexpression of lncRNA NEAT1 promotes VSMCs proliferation and reduces early apoptosis by sponging the miR-490-3p/hnRNPA1 axis [99].

These apoptosis-inhibiting lncRNAs not only maintain fibrous cap structural thickness but also delay AS progression by stabilizing mitochondrial function, blocking caspase cascade reactions, or upregulating anti-apoptotic proteins, providing new potential targets for developing therapeutic approaches based on plaque stabilization strategies.

## 4. LncRNAs and Macrophages

**Figure 3:**
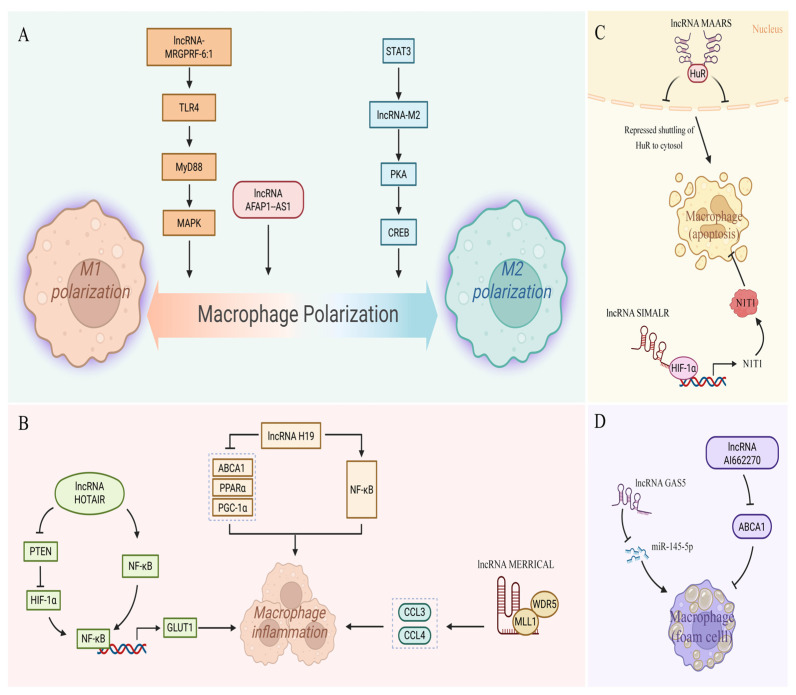
Molecular mechanism of lncRNA regulating macrophage function. In the figure, solid arrows indicate activation or promotion; T-shaped arrows represent inhibition; dashed boxes denote key affected indicators; double wavy lines represent double-stranded DNA. (**A**) Regulation of polarization: lncRNA-MRGPRF-6:1 and lncRNA-AFAP1-AS1 promote pro-inflammatory M1 polarization via activating the TLR4/MyD88/MAPK signaling axis, while the STAT3/lncRNA-M2 axis induces anti-inflammatory M2 polarization through the PKA/CREB pathway. (**B**) Regulation of inflammation: lncRNA HOTAIR drives macrophage inflammation via the PTEN/HIF-1α/NF-κB axis; lncRNA H19 amplifies NF-κB-mediated inflammation by inhibiting the ABCA1/PPARα/PGC-1α axis; lncRNA MERRICAL upregulates the expression of chemokines CCL3 and CCL4 through the MLL1/WDR5 complex, exacerbating inflammatory infiltration. (**C**) Regulation of apoptosis: lncRNA MAARS induces macrophage apoptosis by inhibiting the nucleocytoplasmic shuttling of HuR protein, while lncRNA SIMALR promotes apoptosis via the HIF-1α/NIT1 signaling pathway, participating in inflammatory resolution. (**D**) Regulation of foam cell formation: lncRNA GAS5 acts as a ceRNA to sponge miR-145-5p, and lncRNA Al662270 directly upregulates ABCA1 expression, jointly modulating cholesterol efflux and macrophage foam cell formation.Created in BioRender. Jingyue_Wei. (2026) https://app.biorender.com/illustrations/69be4ddacd6bc4537ef26b17 (accessed on 10 February 2026).

Macrophages exert central, complex roles throughout the occurrence and development of AS, with their functions evolving dynamically from early protective lipid scavenging to late destructive effects [104]. By forming foam cells, amplifying inflammation, and impairing plaque stability, macrophages ultimately become core drivers of AS progression and acute clinical events (e.g., myocardial infarction). Thus, targeting macrophage biological behaviors (polarization, apoptosis, and inflammatory responses) has emerged as a key direction for AS therapeutic research. Notably, long non-coding RNAs (lncRNAs) finely regulate core macrophage functions, including M1/M2 polarization balance, lipid uptake/efflux, inflammatory factor secretion, and apoptosis [105], highlighting the great significance of exploring lncRNA mechanisms in macrophages for AS prevention and treatment (Figure 3, Table 3).

### 4.1. LncRNAs and Macrophage Polarization

As a complex inflammatory vascular disease, AS relies on macrophages’ high plasticity. Macrophages can polarize into functionally distinct phenotypes (predominantly pro-inflammatory M1 and anti-inflammatory M2) in response to different microenvironmental signals [116]. LncRNAs act as central regulators in maintaining the precise balance of this polarization process, directly influencing the inflammatory microenvironment and pathological progression of AS. In positive regulatory mechanisms promoting M2 polarization, investigations have demonstrated that the transcription factor STAT3 enhances lncRNA-M2 transcription and elevates epigenetic histone modification marks within its promoter region, ultimately facilitating M2 macrophage differentiation via the PKA/CREB pathway in LPS-induced AS-related macrophage models [106]. Furthermore, in mechanisms promoting M1 polarization, it was found that inhibiting lncRNA-MRGPRF-6:1 can significantly reduce foam cell formation and decrease M1 macrophage polarization in ox-LDL-induced AS macrophage models. Its function involves regulating macrophage polarization direction through the TLR4-MyD88-MAPK signaling pathway [107]. Similarly, lncRNA AFAP1-AS1 overexpression was also confirmed to promote M1 polarization in LPS- and IFN-γ-treated THP-1 cells (LPS/IFN-γ-induced AS-related macrophage models) while inhibiting M2 polarization [108].

### 4.2. LncRNAs and Macrophage Inflammation

In ox-LDL-elicited macrophage paradigms (ox-LDL-induced AS macrophage models), diverse lncRNAs modulate atherogenesis by orchestrating lipid homeostasis and inflammatory signaling. Knockdown of lncRNA H19 has been confirmed to upregulate ABCA1, PPARα, and its coactivator 1α protein expression, while downregulating NF-κB signaling pathway activity, thereby effectively slowing ox-LDL-induced macrophage inflammation and oxidative stress levels in THP-1-derived AS macrophage models [109]. Additionally, lncRNA MERRICAL was found to have pro-atherogenic effects in ApoE^−/−^ and Ldlr^−/−^ mouse AS models (fed high-fat, high-sugar diets), with this conclusion confirmed in both strains. Mechanistic investigations reveal that MERRICAL-deficient macrophages display attenuated CCL3 and CCL4 expression, concomitant with impaired chemotaxis and inflammatory reactivity, thereby underscoring this lncRNA’s pivotal function in orchestrating macrophage inflammatory responses [110]. LncRNA HOTAIR can regulate metabolic programs in immune cells in LPS-induced AS-related macrophage models; its knockdown can reduce macrophage glucose uptake during LPS-induced inflammation and also regulates expression of other key upstream factors of glucose metabolism, including PTEN and HIF1α, indicating its important role in connecting inflammation and metabolic reprogramming [111].

### 4.3. Other Regulatory Mechanisms in Macrophages

Throughout AS evolution, macrophage-restricted lncRNAs govern cardinal events, including apoptosis and energy metabolism. The macrophage-specific transcript lncRNA-MAARS has been validated as a critical node controlling macrophage cell death and endocytosis; it sequesters the RNA-binding protein HuR/ELAVL1 in the nucleus, thereby preventing its cytoplasmic translocation and modulating apoptotic regulators such as p53 and caspase-9. Notably, MAARS silencing markedly attenuates macrophage apoptosis, an effect that remains independent of systemic lipid parameters or inflammatory milieu [112]. Separately, lncRNA-SIMALR exhibits elevated expression within atherosclerotic lesions in human AS plaque-derived macrophage models; mechanistic hints indicate that SIMALR may engage HIF1α to modulate NTN1 transcription, thus participating in the control of macrophage apoptosis [113].

Beyond apoptosis regulation, lncRNAs also play key roles in macrophage lipid metabolism and foaming. Studies have found that environmental pollutants, phthalates, can increase lipid uptake by downregulating lncRNA GAS5 expression and altering miR-145-5p regulation, thereby exacerbating VSMCs damage and promoting macrophage foam cell formation, ultimately accelerating AS progression [114]. Conversely, lncRNA-AI662270 exerts atheroprotective actions in high-fat diet-induced AS macrophage models; its downregulation potently impedes foam cell formation by amplifying cholesterol efflux and diminishing global intracellular cholesterol burden [115]. Collectively, these data illuminate that discrete lncRNAs critically modulate AS pathogenesis through governing pivotal biological programs, including macrophage apoptosis and lipid homeostasis, thereby furnishing a robust theoretical framework for dissecting AS molecular circuitry and for devising innovative therapeutic paradigms.

## 5. LncRNAs and Lipid Metabolism

Abnormal lipid metabolism represents the core initiating factor in AS occurrence. Elevated blood low-density lipoprotein cholesterol (LDL-C) enters the vascular subintima and undergoes oxidative modification into oxidized low-density lipoprotein (ox-LDL), which is not only cytotoxic and damages endothelial cells but also triggers inflammatory responses, attracting monocyte infiltration into the subintima and differentiation into macrophages. Macrophages internalize massive ox-LDL via scavenger receptors and transdifferentiate into foam cells, with the resultant lipid streaks constituting the earliest histopathological hallmark of AS. LncRNAs critically orchestrate multiple facets of this pathological cascade by regulating lipid metabolism disorders, and their roles exhibit clear functional duality, either promoting or inhibiting AS progression. Below is a systematically reorganized and evidence-stratified discussion based on strictly AS-related research.

LncRNAs that promote lipid metabolism disorders and aggravate AS drive AS progression by disrupting lipid homeostasis, accelerating foam cell formation, and amplifying lipid metabolism disorders, with consistent evidence from AS-specific models (e.g., ox-LDL-induced cells and ApoE^−/−^ mice). Among them, lncRNA-H19 is a key driver extensively involved in AS-related lipid metabolism disorders through multi-model validation. In ox-LDL-induced RAW264.7 macrophages (an AS-specific M1 polarization model), silencing lncRNA H19 inhibits ox-LDL-induced adipogenesis and inflammatory responses by upregulating miR-130b, suggesting that the H19/miR-130b axis represents a potential therapeutic target for AS [117]. As a molecular sponge for miR-let-7a, it alleviates IL-6 suppression and triggers endothelial activation and monocyte recruitment, while reducing high-density lipoprotein (HDL) levels and weakening cholesterol reverse transport by inhibiting apolipoprotein A1 synthesis in hepatocytes [118,119]. In ox-LDL-induced RAW264.7 cells, it competitively binds miR-146a-5p, relieving its inhibition of ANGPTL4 (angiopoietin-like protein 4), upregulating ANGPTL4 expression, leading to lipid accumulation (e.g., LDL-C) in macrophages and promoting foam cell formation [120]. Other pro-atherogenic lncRNAs also play important roles in regulating lipid metabolism: lncRNA-HC forms a complex with hnRNPA2B1 in hepatocytes, inhibiting LXR-mediated cholesterol efflux-related key genes (ABCA1 and Cyp7a1) and reducing cholesterol excretion, thereby disrupting systemic lipid homeostasis and promoting AS [121]; in ApoE^−/−^ mice (classic AS animal model), lncRNA GM47544 regulates apolipoprotein C3 (ApoC3) expression by inducing ubiquitination-dependent protein degradation of APOC3, affecting intracellular triglyceride metabolism, and its high expression leads to hyperlipidemia and dyslipidemia, promoting arterial plaque development [122].

In contrast, lncRNAs that inhibit lipid metabolism disorders and alleviate AS maintain lipid homeostasis, promote cholesterol efflux, and inhibit foam cell formation, with robust evidence from AS-specific models, providing potential therapeutic targets. Other lncRNAs regulate cholesterol synthesis and efflux to exert anti-atherogenic effects: in ApoE^−/−^ mice and hepatocytes, lncRNA-p21 activates the p53 pathway, inhibits the expression of HMG-CoA reductase (rate-limiting enzyme for cholesterol synthesis) in hepatocytes, reduces endogenous cholesterol production, and alleviates AS [95]. LncRNA TUG1 (taurine-upregulated gene 1) acts as a multi-mechanistic regulator of lipid homeostasis: in ApoE^−/−^ mice, it is widely associated with cholesterol efflux genes (ApoM, ABCA1, and ABCG1), and increased TUG1 levels reduce the expression of these genes, lowering cholesterol efflux rate and effectively inhibiting AS progression [123]; in human THP-1 monocytes treated with ox-LDL (AS-specific model), overexpression of lncRNA HOXC-AS1 suppresses ox-LDL-elicited cholesterol accumulation [124]. LncRNA-ANRIL (CDKN2B-AS1) is downregulated in AS plaques and THP-1 macrophage-derived foam cells, and its overexpression attenuates ADAM10 transcription (elevated ADAM10 promotes intracellular cholesterol accrual), enhances cholesterol efflux, and regulates the PPARγ/LXRα signaling pathway to promote ABCA1-mediated cholesterol efflux from macrophages [125,126]; in RAW264.7 cells, lncRNA MeXis amplifies LXR-dependent transcriptional activation of ABCA1 expression, synergistically promoting cholesterol efflux and inhibiting foam cell formation (Table 4) [127].

## 6. Traditional Chinese Medicine Modulates AS by Regulating LncRNA

**Figure 4:**
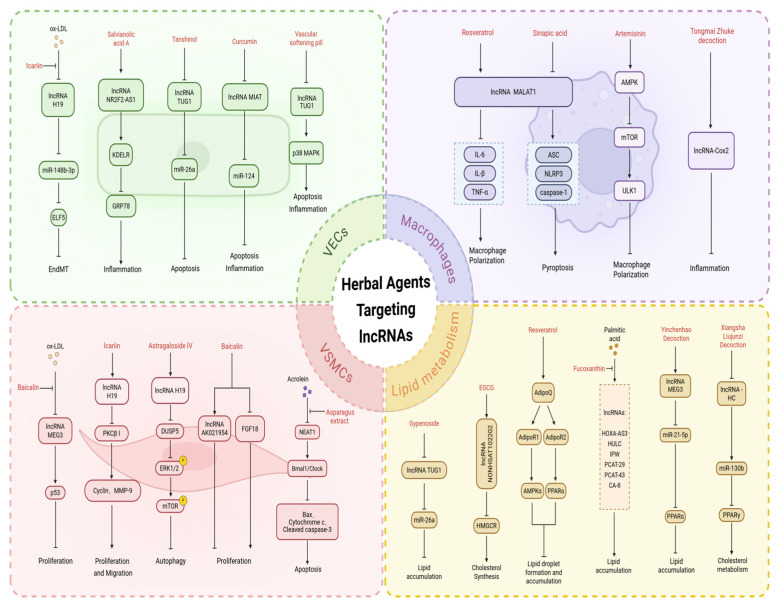
TCM modulates AS by regulating LncRNA: From Cellular Function to Lipid Metabolism. In the figure, solid arrows indicate activation or promotion; T-shaped arrows represent inhibition; dashed boxes denote key affected indicators. This figure summarizes the molecular network of herbal ingredients targeting lncRNAs in the core pathological processes of AS, divided into four modules by cell type and function. VECs: Herbal ingredients (icarilin, salvianolic acid A, tanshinol, curcumin, and vascular softening pill) protect endothelial function by targeting lncRNAs (H19, NR2F2-AS1, TUG1, and MIAT) to inhibit EndMT, inflammation, and apoptosis. VSMCs: Baicalin, icarilin, astragaloside IV, baicalein, and asparagus extract modulate VSMCs proliferation, migration, autophagy, and apoptosis through lncRNAs (MEG3, H19, AK021954, and NEAT1) and their downstream axes. Macrophages: Resveratrol, sinapic acid, artemisinin, and Tongmai Zhuke decoction regulate macrophage polarization, pyroptosis, and inflammation via lncRNA MALAT1, lncRNA-Cox2, and related signaling pathways. Lipid metabolism: Gypenoside, EGCG, resveratrol, fucoxanthin, Yinchenhao Decoction, and Xiangsha Liujunzi Decoction regulate lipid synthesis, accumulation, and cholesterol metabolism by targeting lncRNAs (TUG1, NONHSAT1102202, MEG3, and lncRNA-Hc) and related signaling pathways.Created in BioRender. Jingyue_Wei. (2026) https://app.biorender.com/illustrations/69bf1432fc59b212f6252f81 (accessed on 10 February 2026).

While statins, currently widely used clinically, have demonstrated definite efficacy against AS, their side effects remain non-negligible. Therefore, developing therapeutic agents with higher safety profiles and fewer toxic side effects holds great significance for AS prevention and treatment. Recent investigations have increasingly confirmed that various herbal monomers and compound formulations can intervene in AS progression through multiple pathways by targeting lncRNAs, thereby influencing key targets, including VECs, VSMCs, and macrophages. However, it is important to note that while these studies demonstrate regulation of lncRNAs and associated molecular pathways, direct in vivo evidence linking these specific regulatory events to the attenuation of atherosclerotic plaque development is not always fully established. Much of the current evidence supports a mechanistic association rather than a definitive causal chain from lncRNA modulation to disease modification. Based on this, a systematic categorization and discussion of how herbal monomers and compound formulations regulate cellular functions by targeting lncRNAs is provided, with careful distinction between mechanistic findings and validated anti-atherosclerotic efficacy (Figure 4, Table 5).

### 6.1. Regulation of VECs by Herbal Monomers and Compound Formulations Through Targeting LncRNAs

Research has shown that various TCM monomers and compound formulations exhibit clear protective effects on VECs. Icariin serves as a typical example. Icariin, a principal bioactive constituent of the traditional tonic herb Epimedium, has recently been shown to provide significant cardiovascular protection and attenuate the development of AS. Accumulated evidence confirms that icariin effectively mitigates oxidized low-density lipoprotein-evoked vascular endothelial cell injury and attenuates atherosclerotic lesion development in high-fat diet-fed ApoE^−/−^ murine models. Its mechanism is closely related to ferroptosis alleviation through autophagy promotion [13]. In a 12-week intervention trial, icariin markedly diminished aortic plaque burden in ApoE^−/−^ mice. Via high-throughput sequencing coupled with bioinformatic interrogation of aortic tissue, investigators systematically delineated the putative functionalities of icariin-responsive differentially expressed lncRNAs by GO and KEGG enrichment analyses, thereby establishing a critical platform for subsequent identification of novel therapeutic targets against AS [143]. Endothelial-to-mesenchymal transition (EndMT), whereby VECs relinquish their endothelial signature and gain mesenchymal traits, critically underpins AS initiation and progression, and is tightly controlled by lncRNAs [144]. Liu et al. first delineated the lncRNA-H19/miR-148b-3p/ELF5 axis as a pivotal regulator of ox-LDL-elicited EndMT in human umbilical vein endothelial cells and ApoE^−/−^ mice, and verified that icariin efficiently suppresses EndMT via modulation of this pathway. This suggests a potential mechanism through which icariin may confer anti-atherosclerotic protection, although direct evidence linking H19-mediated EndMT suppression to plaque reduction requires further investigation [128]. These studies systematically elucidate, at different levels, the multiple mechanisms by which icariin protects VECs and may inhibit AS progression by regulating lncRNA-related signaling pathways, providing new theoretical bases and experimental evidence for AS prevention and treatment by active TCM components. Similarly, active components from Salvia miltiorrhiza (Danshen) also demonstrate significant endothelial protective effects. Salvianolic acid A is one of the main water-soluble active components of Danshen. Studies have found that it can inhibit the lncRNA NR2F2-AS1-KDELR signaling axis in VECs, thereby reducing endoplasmic reticulum chaperone glucose-regulated protein 78 (GRP78) secretion [15]. Another important Danshen active component, Danshensu, has been confirmed to inhibit endothelial cell apoptosis and subsequently regulate the lncRNA TUG1/miR-26a axis [16]. This suggests that the aforementioned active components of Salvia miltiorrhiza (Danshen) have the potential to regulate AS, but whether they can delay the progression of AS remains to be further investigated.

Meanwhile, other TCM monomers also have unique characteristics in endothelial protection. Curcumin is a flavonoid chiefly sourced from Curcuma longa. It demonstrates a broad profile of pharmacological activities, encompassing antioxidant, anti-inflammatory, hepatoprotective, anti-atherosclerotic, and hypoglycemic properties [145]. Evidence demonstrates that curcumin mitigates inflammatory cascades within murine AS models and ox-LDL-stimulated HUVECs, and modulates vascular endothelial apoptosis and proliferation via lncRNA-MIAT-mediated relief of epigenetic repression on miR-124 [17]. Regarding TCM compound formulations, the vessel-softening pill (Xueguan Ruanhua Wan), a formula derived from Baohe Wan under the guidance of the “resolving phlegm and removing blood stasis” principle, is effective in preventing and treating AS. Its molecular mechanism for preventing and treating AS may be related to targeting lncRNA-TUG1 regulation to inhibit p38MAPK signaling pathway activation and its downstream signals T-p38 and p-p38 expression, thereby suppressing vascular inflammatory responses, regulating blood lipids, inhibiting VEC apoptosis, and protecting the vascular endothelium [129].

### 6.2. Regulation of VSMCs by Herbal Monomers and Compound Formulations Through Targeting LncRNAs

In restraining aberrant proliferation, migration, and apoptosis of VSMCs, TCM monomers and composite formulas also exert pivotal functions. The regulatory actions of icariin on VSMCs engage intricate signaling networks; investigations have revealed that ICA inhibits proliferation and migration of HA-VSMCs, altering the expression of cyclin D1 and MMP-9, in a lncRNA H19/HuR-dependent manner [14]. While this demonstrates a clear lncRNA-dependent mechanism in vitro, the contribution of this specific axis to icariin’s anti-atherosclerotic effects in vivo remains to be established. Baicalin is one of the most abundant flavonoids in *Scutellaria baicalensis* Georgi dried roots, with numerous studies showing its extensive pharmacological activities. Research has found that baicalin can reverse upregulated cell cycle-related protein expression in HA-VSMCs induced by lncRNA MEG3 knockdown. Its mechanism likely involves inhibiting cell proliferation and promoting apoptosis via MEG3/p53 signaling pathway modulation, thereby potentially playing roles in AS [130]. Furthermore, other studies have confirmed lncRNA AK021954 and FGF18 involvement in baicalin VSMCs proliferation inhibition processes, although precise molecular mechanisms remain to be fully elucidated [131].

Astragaloside IV suppresses VSMCs autophagy and pathological mineralization, which are mechanisms plausibly linked to lncRNA-H19- and DUSP5-mediated ERK signaling transmission [132]. Asparagus extract, used as a dietary supplement, shows VSMCs protective potential. Research found that in acrolein-induced VSMCs apoptosis models, cell apoptosis was associated with lncRNA NEAT1 and Bmal1/Clock gene downregulation, and asparagus extract could protect VSMCs from this damage, offering a potential mechanism for cardiovascular protection that warrants further in vivo investigation [133].

### 6.3. Regulation of Macrophages by Herbal Monomers and Compound Formulations Through Targeting LncRNAs

In regulating macrophage function, TCM monomers and compound formulations have also demonstrated remarkable efficacy. Extracted from the TCM herb Qinghao (*Artemisia annua* L.), artemisinin is a sesquiterpene lactone that serves as a first-line antimalarial drug [146]. Studies have found that beyond antimalarial effects, artemisinin also possesses excellent anti-inflammatory activity [147,148]. Previous research suggests that artemisinin may help prevent early AS development through pathways such as endothelial function improvement [149]. More importantly, further network pharmacology analysis revealed that during artemisinin AS treatment, 102 differentially expressed lncRNAs are involved. KEGG and GO analyses indicated that these lncRNAs are enriched in metabolic processes and the PI3K-Akt signaling pathway. Among these, lncRNA ENSMUST00000099676.4 was predicted as a potential key molecule involved in artemisinin AS alleviation [134]. This bioinformatic prediction provides a basis for future mechanistic studies, but requires experimental validation to confirm a functional role. Resveratrol, a natural plant polyphenol found in grapes, berries, and other plants, has been proven beneficial for cardiovascular health. It primarily exerts anti-AS effects through anti-inflammatory actions and foam cell formation inhibition [150,151]. Emerging evidence indicates that resveratrol impedes macrophage polarization through modulation of lncRNA-MALAT1, thereby governing the expression of pivotal inflammatory cytokines IL-6, IL-1β, and TNF-α. This suggests a mechanistic pathway that may contribute to its anti-atherosclerotic efficacy [135]. Sinapic acid, a naturally abundant phenolic antioxidant, has been shown to chronically suppress serum endothelin-1 and IL-1β titers while attenuating pyroptotic executors ASC, NLRP3, and caspase-1 under low-dose regimens, and its specific mechanism likely involves macrophage pyroptosis alleviation by downregulating lncRNA MALAT1 in diabetic AS rat models [136]. Furthermore, Zhang et al. identified 58 downregulated lncRNAs in carotid atherosclerotic plaque samples, among which lncRNA-Cox2 was the most significantly downregulated. Tongmai Zhuke Decoction can inhibit macrophage inflammatory responses in carotid AS by upregulating lncRNA-Cox2 [18].

### 6.4. Regulation of Lipid Metabolism by Herbal Monomers and Compound Formulations Through Targeting LncRNAs

Studies have found that certain TCM monomers and compound formulations may exert anti-AS effects by modulating lipid metabolism. Gypenosides, the principal bioactive constituents of the *Cucurbitaceae* species *Gynostemma pentaphyllum*, exhibit anti-tumor, neuroprotective, and anti-atherosclerotic properties, positioning them as candidates with marked translational potential [152]. Mechanistic studies reveal that gypenosides mitigate hepatic lipid deposition in ApoE^−/−^ AS mice via modulation of the lncRNA-TUG1/miR-26a axis and consequent interference with mitochondrial apoptotic cascades, representing a putative pathway that may underpin their anti-atherogenic efficacy [137]. Among green tea active components, epigallocatechin gallate (EGCG), as the most active tea polyphenol molecule, not only directly regulates key lipid metabolism pathway gene expression (such as LDLR, HMGCR, and ACAT2) but also significantly modulates various lncRNA expression levels. These multi-faceted regulatory actions are associated with its effects on AS progression, though the specific contribution of lncRNA modulation remains to be fully delineated [138]. Regarding TCM compound formulation research, Xiangsha Liujunzi Tang (Costusroot and Amomum Six Gentlemen Decoction) influences lipid metabolism and may prevent AS occurrence and development by affecting PPARγ-mediated cholesterol metabolism processes via lnc-HC modulation [139].

Current studies have shown that certain TCM monomers and compound formulations can regulate lipid metabolism through lncRNA modulation in non-AS-specific models. For example, resveratrol impedes lipid-droplet formation and accrual in vivo and in 3T3-L1 preadipocytes via AdipoQ–AdipoR1–AMPKα and AdipoQ–AdipoR2–PPARα cascades [140]. Highly stable fucoxanthin derived from brown algae significantly inhibits lipid accumulation in adipose-derived stem cells under palmitic acid treatment and can reverse their lipid metabolism-related gene expression. Genetic regulation targeting lncRNAs can modulate adipogenesis, suggesting a potential epigenetic mechanism for fucoxanthin that requires further validation [141]. Yinchenhao Tang (Artemisia scoparia decoction), a classical heat- and dampness-purging formula, alleviates metabolic-associated fatty liver disease by potentiating fatty-acid β-oxidation via lncRNA-MEG3-mediated modulation of the miR-21-5p/PPARα axis [142]. However, because these studies were not conducted specifically in AS models, whether they exert potential anti-atherosclerotic effects through regulation of lipid metabolism remains to be further validated.

## 7. Summary and Outlook

LncRNAs, as key regulatory factors in AS pathogenesis, are increasingly demonstrating their research value. Current studies indicate that lncRNAs play significant regulatory roles in core AS processes, such as maintaining VECs function, modulating VSMCs phenotypic switching, and regulating macrophage inflammatory responses, through various molecular mechanisms, including the ceRNA network (the lncRNA-miRNA-mRNA axis), transcription factor complex formation, and epigenetic modification participation. However, significant limitations remain in current research. Most findings are verified only in single cell line experiments, lacking confirmation in animal models within complex physiological environments, and further support from clinical research data. In particular, in TCM research, although several TCM monomers, including icariin, baicalin, and astragaloside IV, have been shown to elicit anti-atherosclerotic effects via specific lncRNAs, the field of TCM research in this area remains in its nascent stage. The high complexity and heterogeneity of TCM lncRNA regulatory networks, whose specific targets and signaling pathways remain poorly elucidated, severely restrict their clinical translation.

Notably, it is crucial to clarify the differences between TCM and internationally recognized guideline-based standard therapies for AS, as well as their potential interactions (synergy, enhancement, or inhibition), which is a key focus of clinical practice and research. Internationally recognized standard therapies for AS, as recommended by guidelines such as the 2019 ESC/EAS Guidelines for the management of dyslipidemias: lipid modification to reduce cardiovascular risk [153], and the 2023 AHA/ACC/ACCP/ASPC/NLA/PCNA Guideline for the Management of Patients With Chronic Coronary Disease: A Report of the American Heart Association/American College of Cardiology Joint Committee on Clinical Practice Guidelines [154], mainly focus on “targeted intervention of pathogenic factors and lesion progression”. Their core measures include lipid-lowering (statins as first-line drugs, supplemented by PCSK9 inhibitors, ezetimibe, etc.), and anti-platelet aggregation (aspirin, clopidogrel, etc.), blood pressure control, blood glucose regulation, and lifestyle intervention. The characteristics of standard therapy are clear targets, single-component drugs, and emphasis on the control of objective indicators (such as LDL-C, blood pressure, and blood glucose) and hard cardiovascular endpoints (myocardial infarction, stroke, and death). In contrast, TCM treats AS based on the holistic concept and syndrome differentiation, which is fundamentally different from the disease-centered standard therapy. TCM holds that AS belongs to the category of “obstruction of collaterals” and “blood stasis” in traditional theory, and its pathogenesis is closely related to qi deficiency, blood stasis, phlegm turbidity, and toxin accumulation [155]. Therefore, TCM intervention focuses on “regulating the overall function of the body” rather than single-target intervention, and often adopts multi-component, multi-target treatment strategies (such as TCM monomers, classic compound prescriptions, and proprietary Chinese medicines) to adjust the balance of qi, blood, and body fluids, thereby improving the body’s resistance to pathogenic factors and intervening in the pathological process of AS [156].

In terms of interaction between TCM and standard therapy, current evidence suggests that the two are not mutually exclusive but complementary, mainly presenting synergistic and enhancing effects, with no obvious inhibitory effects reported. Standard therapy plays a dominant role in quickly controlling key pathogenic factors (such as hyperlipidemia and platelet aggregation) and reducing acute cardiovascular events, while TCM can make up for the limitations of standard therapy in regulating the overall body state, improving clinical symptoms, and reducing long-term complications. Specifically, TCM can enhance the efficacy of standard therapy through multi-target regulation: for example, TCM monomers such as berberine can further reduce blood lipid levels (TC, TG, and LDL-C) on the basis of statin treatment, and regulate the intestinal flora-TMAO axis to enhance lipid-lowering and anti-AS effects [157,158]; proprietary Chinese medicines such as Tongxinluo Capsule can stabilize atherosclerotic plaques and reduce vascular inflammation on the basis of standard lipid-lowering and anti-platelet therapy, thereby reducing the risk of plaque rupture [159,160]. In addition, TCM can also alleviate the adverse reactions of standard therapy (such as statin-induced myalgia and liver function damage), improve patient compliance, and lay a foundation for long-term standardized treatment [161,162]. Regarding potential inhibitory interactions, no clear clinically significant inhibition has been reported to date in the context of AS treatment; however, theoretical concerns regarding potential competition for metabolic pathways—such as potential drug–drug interactions mediated by cytochrome P450 enzymes—warrant attention in future pharmacokinetic studies [163].

Importantly, a large number of randomized controlled trials (RCTs) and meta-analyses have confirmed that incorporating TCM into AS standard therapy can bring significant additional clinical benefits. Landmark evidence comes from two RCTs of Tongxinluo Capsule: the CAPITAL study [159], a multicenter RCT involving 1212 patients with non-calcified carotid plaques, demonstrated that adding Tongxinluo for 24 months significantly reduced carotid plaque area, slowed intima-media thickness progression, and reduced major cardiovascular events from 13.2% to 7.7%. The TXL-CAP study further showed that in patients with acute coronary syndrome, 12-month Tongxinluo add-on therapy significantly increased fibrous cap thickness, reduced maximum lipid arc, and lowered new vulnerable plaque incidence from 10% to 1.3% [160]. In addition, multiple meta-analyses have shown that TCM combined with standard therapy can further improve blood lipid profiles, reduce inflammatory markers (IL-6, TNF-α, and hs-CRP), improve IMT and crouse plaque scores, and reduce the risk of major adverse cardiovascular events (RR = 0.53, 95% CI: 0.40–0.69, *p* < 0.00001), with good safety and tolerability—specifically, no significant increase in adverse events compared with standard therapy alone, which is consistent with TCM’s role in alleviating standard therapy-related side effects [164].

Future research needs to focus on addressing the following key issues. First, more comprehensive animal model validation systems should be established, utilizing classic models such as ApoE^−/−^ mice combined with high-fat diets for in-depth validation. Second, advanced technologies like single-cell sequencing and spatial transcriptomics should be employed to systematically analyze the dynamic changes in lncRNA expression profiles under TCM intervention. Furthermore, lncRNA structure and function research should be strengthened to clarify direct interaction mechanisms between lncRNAs and TCM active components. Notably, with gene editing technology and nucleic acid drug development breakthroughs, TCM-derived therapeutic strategies targeting specific lncRNAs show promising prospects. In the future, based on TCM multi-component, multi-target characteristics, lncRNA-TCM interaction network maps can be constructed to provide theoretical foundations for precise medication. Simultaneously, developing TCM-based nano-formulations targeting pathogenic lncRNAs could enhance treatment specificity. Additionally, clinical translational research on TCM lncRNA regulation should be promoted to establish individualized treatment plans.

In summary, although TCM AS prevention and treatment research through lncRNA regulation faces challenges, prospects are broad. By integrating modern molecular biology technologies, bioinformatics methods, and traditional TCM theory, it is promising to develop innovative TCM preparations based on lncRNA targets on the foundation of mechanism of action elucidation, thereby providing new strategies and methods for AS prevention and treatment. With continuous research depth and breadth expansion, this field is destined to open new avenues for cardiovascular disease prevention and treatment.

## Figures and Tables

**Table 1:** The Regulatory Role and Mechanism of lncRNAs in VECs Under Atherosclerotic and Vascular Injury Conditions.

Effect	lncRNA	Targeted miRNA	Model	Context	Molecular Mechanism	References
Pro-apoptotic	lncRNA PVT1	---	HUVECs	AS-specific (ox-LDL-induced)	ERK1/2 ↑, p38 ↑, promotes ox-LDL-induced HUVECs apoptosis	[29]
	lncRNA TP73-AS1	miR-654-3p	HAECs	AS-specific (ox-LDL-induced)	lncRNA TP73-AS1 targets the miR-654-3p/AKT3 axis, miR-654-3p ↓, AKT3 ↑, promotes ox-LDL-induced endothelial cell apoptosis	[30]
	lncRNA-ASLNC18810	miR-559	ARECs	AS-specific (vascular endothelial dysfunction in AS)	lncRNA-ASLN18810 targets the HIF-1α/miR-559/Bax axis, miR-559 ↓, HIF-1α ↑, indirectly positively regulates post-transcriptional Bax, promoting ECs apoptosis	[31]
	lncRNA DANCR	miR-214-5p	VSMCs, HUVECs	AS-specific (AS development)	Targets miR-214-5p/COX20, IL-6, IL-1β, TNF-α, MDA ↑, promotes VSMCs apoptosis	[32]
	lncRNA HOXA11-AS	miR-515-5p	ApoE^−/−^ mice	AS-specific (ApoE^−/−^ mouse model, ox-LDL-induced)	Directly inhibits miR-515-5p, ROCK1 ↑, eNOS ↓, promotes ox-LDL-induced HUVECs injury	[33]
	lncRNA SNHG12	miR-218-5p	HUVECs	AS-specific (ox-LDL-induced)	Enhances ox-LDL-induced injury and promotes apoptosis via the miR-218-5p/IGF2 axis	[34]
	lncRNA-TUG1	miR-148b	HUVECs	AS-specific (ox-LDL-stimulated)	lncRNA-TUG1 targets the miR-148b/IGF2 axis, miR-148b ↑, IGF2 ↓, alleviates ox-LDL-induced HUVECs apoptosis and injury	[35]
	lncRNA AK087124	miR-224-5p	MAECs, C57BL/6J mice	AS-specific (ox-LDL-induced)	Endothelial cell apoptosis and inflammation are regulated by the lncRNA AK087124/miR-224-5p/PTEN axis through the AKT signaling pathway; silencing miR-224-5p and overexpressing PTEN reverse AK087124’s effect on ox-LDL-induced injury	[36]
	lncRNA OIP5-AS1	miR-98-5p	HUVECs	AS-specific (ox-LDL-induced)	Downregulates miR-98-5p via the TLR4/NF-κB signaling pathway, mediates HMGB1 expression, thereby promoting apoptosis	[37]
Anti-apoptotic	lncRNA NORAD	---	HUVECs, ApoE^−/−^ mice	AS-specific (ApoE^−/−^ mouse model, ox-LDL-induced)	NF-κB ↓, p53-p21 ↓, IL-8 ↓, alleviates HUVECs senescence and apoptosis	[38]
	lncRNA FGF7-5/GLRX3	miR-2681-5p	HUVECs	AS-specific (ox-LDL-induced)	lncRNA FGF7-5 and lncRNA GLRX3 jointly target miR-2681-5p, ERCC4 ↑, reduce HUVECs apoptosis and carotid plaque formation	[39]
	lncRNA MIR4697HG	---	HUVECs	AS-specific (aox-LDL-induced)	lncRNA MIR4697HG alleviates ox-LDL-induced HUVECs apoptosis, oxidative stress, and adhesion molecule release; FUS ↑, ANXA5 ↓, alleviates AS progression in mice	[40]
pyroptotic	lncRNA MALAT1	miR-22	EA.hy926, HUVECs	AS-specific (high glucose-induced, a key AS risk factor)	lncRNA MALAT1 competitively binds miR-22, NLRP3 ↑, regulates high glucose-induced HUVECs pyroptosis	[41]
	lncRNA NEAT1	---	VECs, NEAT1 knockout mice	AS-specific (endothelial pyroptosis in AS)	Binds KLF4, NLRP3 ↑, triggers VECs pyroptosis; Exercise can downregulate NEAT1 expression via m6A epigenetic modification, thereby inhibiting endothelial pyroptosis to slow AS progression	[42]
	lncRNA Gaplinc	---	ApoE^−/−^ mice, endothelial cells	AS-specific (ApoE^−/−^ mouse model, ox-LDL-induced)	Silencing lncRNA Gaplinc inhibits its interaction with SP1, NLRP3 ↓, thereby alleviating endothelial cell pyroptosis and AS plaques in high-fat diet-fed mice	[43]
	lncRNA MEG3	miR-223	HAECs, ApoE^−/−^ mice	AS-specific (ApoE^−/−^ mouse model)	Inhibits miR-223 function, NLRP3 ↑, promotes HAECs pyroptosis	[44,45]
	lncRNA RP11-490M8.1	---	HUVECs	Vascular injury (LPS-induced, inflammatory vascular damage)	lncRNA RP11-490M8.1 inhibits LPS-induced expression of inflammatory factors (IL-6, TNF-α, NF-κB)	[46]
Proliferation/Migration	lncRNA HOTAIRM1	---	HUVECs	Vascular injury (ox-LDL-induced)	HOTAIRM1 interacts with HOXA4 ↑; HSPA5 overexpression partially antagonizes HUVEC proliferation inhibition upon HOTAIRM1 depletion	[47]
	lncRNA RNCR3	miR-185-5p	VECs	AS-related (inflammatory cytokine secretion in ECs)	Drives cell cycle progression via the miR-185-5p/cyclin D2 axis, IL-6, IL-1β, TNF-α ↑, promotes VECs proliferation	[48]
	lncRNA SNHG7	miR-186-5p	HUVECs	AS-specific (ox-LDL-induced)	miR-186-5p binds both the SNHG7 3′-UTR and the MMP2 mRNA 3′-UTR; by binding to the promoter region of lncRNA SNHG7, transcription factor E2F1 upregulates its expression	[49]
Anti-proliferation/Migration	lncRNA ZFAS1	---	HUVECs, VECs	AS-specific (ox-LDL-induced EndMT)	Upregulating lncRNA ZFAS1 decreases inflammatory factors IL-1β, IL-6, TNF-α ↓, and affects the Wnt/β-catenin signaling pathway to regulate HUVECs proliferation	[50]
	lncRNA TUG1	---	HUVECs, Wistar rats	AS-specific (metformin-mediated anti-AS)	Metformin exerts anti-AS effects by targeting lncRNA TUG1 to activate the AMPK/mTOR signaling pathway	[51]
	lncRNA-TUG1	---	HUVECs	Vascular injury (ox-LDL)	promoted vascular injury repairing in vivo via the Runx2/ANPEP axis	[52]
	lncRNA XXYLT1-AS2	---	HUVECs	AS-specific (inflammatory response in AS)	Overexpression of lncRNA XXYLT1-AS2 decreases VCAM-1 and MCP-1 ↓, directly interacts with target genes FUS/cyclin D1 to regulate ECs proliferation and migration; NF-κB ↓, exerts protective effects against inflammatory response in AS	[53]
Other	lncRNA AF131217.1	miR-128-3p	HUVECs	Vascular injury (laminar shear stress-induced)	Laminar shear stress-regulated lncRNA AF131217.1 inhibits ICAM-1 and VCAM-1 expression via the AF131217.1/miR-128-3p/KLF4 axis	[54]
	lncRNA AK136714	---	ApoE^−/−^ mice, HUVECs	AS-specific (ApoE^−/−^ mouse model)	lncRNA AK136714 can impair the endothelial barrier and increase inflammatory response and apoptosis in HUVECs	[55]
	lncRNA MALAT1	miR-181b	HUVECs	AS-specific (ox-LDL-induced)	lncRNA MALAT1 enhances the effect of miR-181b, TOX ↑, making endothelium susceptible to ox-LDL-induced endothelial cell inflammation and oxidative stress	[56]
	lncRNA VINAS	---	LDLR^−/−^ mice, ECs, SMCs	AS-specific (LDLR^−/−^ mouse model)	lncRNA VINAS mediates inflammatory pathways by regulating NF-κB and p38 MAPK signaling pathways to regulate AS	[57]
	lncRNA-GAS5	miR-193-5P	Human aortic endothelial cells	AS-specific (atherogenesis via impaired endothelial autophagy)	Overexpression of lncRNA-GAS5 inhibits endothelial cell autophagy and autophagic vacuole accumulation via the miR-193-5P/SRSF10 signaling pathway	[58]
	lncRNA FENDRR	miR-18a-5p	HAECs	AS-specific (ox-LDL-induced)	FENDRR reverses ox-LDL-induced mitochondrial energy metabolism disorder by regulating miR-18a-5p ↓ or PGC-1α ↑	[59]

↑ indicate promotion; ↓ represent inhibition.

**Table 2:** The Regulatory Role and Mechanism of lncRNAs in VSMCs Under Atherosclerotic and Vascular Injury Conditions.

Effect	lncRNA	Targeted miRNA	Model	Context	Molecular Mechanism	References
phenotypic switching	lncRNA-ANRIL	---	VSMCs	AS-specific (ox-LDL-induced, AS plaque formation)	Metformin administration increases lncRNA-ANRIL levels, inhibits VSMCs phenotypic switching, and prevents AS plaque formation	[67]
	lncRNA PEBP1P2	---	SD rats, VSMCs	AS-specific (SD rat model, ox-LDL-induced)	lncRNA PEBP1P2 inhibits VSMCs phenotypic switching, abnormal proliferation, and migration by targeting the CDK9 pathway	[68]
	lncRNA NEAT1	---	VSMCs	AS-specific (ox-LDL-induced, VSMCs phenotypic switching)	When lncRNA NEAT1 is lowly expressed, released WDR5 opens the promoter regions of VSMCs contractile-related genes to promote their expression	[69]
	lncRNA NEAT1	---	VSMCs, ApoE^−/−^ mice	AS-specific (high-fat diet-induced, VSMCs osteogenic differentiation)	lncRNA NEAT1 functions in AS by regulating the epigenetic function of EZH2, thereby enhancing VSMCs proliferation, migration, and osteogenic differentiation	[70]
Proliferation/Migration	lncRNA ANRIL	miR-399-5p	HA-VSMCs, HUVECs	AS-specific (ox-LDL-induced)	lncRNA ANRIL sponges miR-399-5p, regulates the RAS/RAF/ERK signaling pathway, thereby promoting ox-LDL-induced HA-VSMCs proliferation and migration	[71]
	lncRNA-XIST	miR-539-5p	VSMCs	AS-specific (ox-LDL-induced)	lncRNA-XIST acts as a ceRNA for miR-539-5p, promotes ox-LDL-stimulated VSMCs proliferation and migration via the miR-539-5p/SPP1 axis	[72]
	lncRNA XIST	miR-761	HVSMCs	AS-specific (ox-LDL-induced, cerebral AS)	Knockdown of lncRNA XIST targets inhibition of the BMP9/ALK1/endoglin pathway, inhibits HVSMCs proliferation and migration by promoting miR-761	[73]
	lncRNA SNHG16	miRNA-22-3p	VSMCs	AS-specific (ox-LDL-induced)	lncRNA SNHG16 acts as a ceRNA for miRNA-22-3p, accelerates AS plaque formation and enhances ox-LDL-activated VSMCs proliferation and migration by targeting the miRNA-22-3p/HMGB2 axis	[74]
	lncRNA SNHG16	miR-30c-5p	VSMCs	AS-specific (ox-LDL-induced, cerebral AS)	Downregulation of lncRNA SNHG16 inhibits activation of the MEK/ERK signaling pathway and inhibits ox-LDL-induced VSMCs proliferation and migration by targeting the miR-30c-5p/SDC2 axis	[75]
	lncRNA MIAT	miR-326	VSMCs	AS-specific (ox-LDL-induced, AS progression)	lncRNA MIAT competitively sequesters miR-326 and promotes AS progression; upregulating miR-326 reverses MIAT-mediated promotion of VSMCs migration	[76]
	lncRNA PUNISHER	---	ApoE^−/−^ mice, HA-VSMCs	AS-(ApoE^−/−^ mouse model, ox-LDL-induced)	PUNISHER expression promotes cell proliferation, migration, and promotes VSMCs mitochondrial fission and activation of various apoptosis-related proteins	[77]
	lncRNA AL355711	---	VSMCs	AS-specific (ox-LDL-induced, VSMCs migration)	lncRNA AL355711 promotes VSMCs migration via the ABCG1/MMP3 pathway	[78]
	lncRNA BANCR	miR-34c	HASMCs	AS-specific (ox-LDL-induced)	Overexpression of lncRNA BANCR promotes HASMCs proliferation in AS through a mechanism involving miR-34c hypomethylation and subsequent reversal of miR-34c-mediated suppression on HMGB1, TNF-α, and Bcl-2	[79]
	lncRNA-ITGA2	---	HA-VSMCs	AS-specific (ox-LDL-induced, vascular remodelling)	lncRNA-ITGA2 directly binds its complementary sequence in the nucleus and increases ITGA2 transcriptional activity, synergistically activating the ITGA2-FAK signaling pathway to promote HA-VSMCs proliferation/migration	[80]
	lncRNA OIP5-AS1	miR-141-3p	VSMCs	AS-specific (ox-LDL-induced)	lncRNA OIP5-AS1 indirectly increases HMGB1 expression in VSMCs by targeting miR-141-3p, promotes VSMCs proliferation, migration, and inhibits apoptosis	[81]
	lncRNA 430945	---	VSMCs	AS-Related (Ang II-induced, AS risk factor)	Downregulation of lncRNA 430945 significantly inhibits Ang II-induced VSMCs proliferation and migration	[82]
	lncRNA PVT1	---	HA-VSMCs	AS-specific (ox-LDL-induced)	Silencing lncRNA PVT1 inhibits HA-VSMCs proliferation and promotes apoptosis by downregulating the MAPK/NF-κB pathway	[83]
	lncRNA TUG1	miR-21	ApoE^−/−^ mice, HA-VSMCs	AS-specific (ApoE^−/−^ mouse model, ox-LDL-induced)	lncRNA TUG1 competes with PTEN for binding miR-21, promotes VSMCs proliferation and induces apoptosis in vitro	[84]
Anti-proliferation/Migration	lncRNA MEG3	miR-361-5p	VSMCs	AS-specific (ox-LDL-induced)	lncRNA MEG3 acts as a ceRNA for miR-361-5p, regulates ABCA1 expression involved in VSMCs proliferation; inhibiting MEG3 promotes ox-LDL-induced VSMCs proliferation	[85]
	lncRNA UCA1	miR-26a	VSMCs	AS-specific (ox-LDL-induced, anti-AS regulation)	lncRNA UCA1 downregulates miR-26a expression, thereby relieving inhibition of its target gene PTEN, inhibiting VSMCs abnormal proliferation against AS	[86]
	lncRNA LEF1-AS1	miR-544a	VSMCs	AS-specific (ox-LDL-induced)	Overexpression of miR-544a reverses the inhibitory effect of lncRNA LEF1-AS1 on VSMCs proliferation and migration, mediated via the PTEN pathway	[87]
	lncRNA-SNHG7-003	miR-1306-5p	VSMCs	AS-specific (ox-LDL-induced, VSMCs invasion)	LncRNA-SNHG7-003 exerts inhibitory effects on VSMCs proliferation, migration, and invasion via the miR-1306-5p/SIRT7 signaling pathway	[88]
	lncRNA TPRG1-AS1	---	HASMCs	AS-specific (ApoE^−/−^ mouse model, ox-LDL-induced)	lncRNA TPRG1-AS1 directly binds MYH9 protein, their interaction promotes MYH9 degradation via the proteasome pathway, hinders F-actin stress fiber formation, ultimately inhibiting HASMCs migration	[89]
	lncRNA RP4-639F20.1	---	ApoE^−/−^ mice, VSMCs	AS-specific (ApoE^−/−^ mouse model, high-fat diet-induced)	lncRNA RP4-639F20.1 interacts with THRAP3, regulates the c-FOS pathway, decreases MMP10 and VEGF-α in VSMCs, and inhibits their proliferation and migration	[90]
	lncRNA ZNF800	---	VSMCs	AS-specific (ox-LDL-induced)	Overexpression of lncRNA ZNF800 inhibits AKT/mTOR/HIF-1α signaling pathway activity, VEGF-α and MMP1 ↓, inhibits VSMCs proliferation and migration	[91]
	lncRNA NUDT6	---	SMCs	Vascular injury (high-fat diet-induced, vascular disease progression)	Silencing lncRNA NUDT6 triggers SMCs survival and migration	[92]
	lncRNA AC105942.1	---	VSMCs	Vascular injury (Ang II-induced, AS risk factor)	Downregulates hnRNPA2B1, thereby inhibiting Ang-II-induced VSMCs proliferation	[93]
Pro-apoptotic	lncRNA DANCR	miR-214-5p	VSMCs	AS-specific (ox-LDL-induced)	Acts as a ceRNA targeting the miR-214-5p/COX20 axis; its downregulation reduces apoptosis, increases viability of ox-LDL-treated VSMCs, promoting AS progression	[32]
	lncRNA CASC11	---	VSMCs	AS-specific (ox-LDL-induced)	Overexpression of lncRNA CASC11 may improve AS by downregulating IL-9, inhibiting VSMCs proliferation, and promoting apoptosis	[94]
	lncRNA-p21	---	ApoE^−/−^ mice, HA-VSMCs	AS-specific (ApoE^−/−^ mouse model, high-fat diet-induced)	lncRNA-p21 regulates neointima formation, inhibits VSMCs proliferation, and induces apoptosis by enhancing p53 activity	[95]
	lncRNA-P21	---	SD rats, BVSMCs	Vascular injury (SD rat model, thoracic aortic aneurysm)	Overexpression of lncRNA-p21 inhibits the proliferation of VSMCs and promotes their apoptosis, while TGF-β1 inhibitors alleviate these effects	[96]
Anti-apoptotic	lncRNA H19	---	ApoE^−/−^ mice, VSMCs	AS-specific (ApoE^−/−^ mouse model, high-fat diet-induced)	Knockout of lncRNA H19 may increase p53-mediated VSMCs apoptosis and reduce abnormal proliferation, thereby alleviating AS deterioration	[97]
	lncRNA Punisher	miR-664a-5p	Sprague-Dawley rats VSMCs	AS-specific (carotid artery model, patients with AS)	Directly binds miR-664a-5p targeting OPA1; Punisher overexpression significantly inhibits neointima formation and VSMCs apoptosis in vivo	[77]
	lncRNA RP11-714G18.1	---	HUVECs, HA-VSMCs	AS-specific (ApoE^−/−^ mouse model, AS plaque formation)	lncRNA RP11-714G18.1 targets and promotes LRP2BP expression, downregulates MMP1 thereby inhibiting HA-VSMCs apoptosis	[98]
	lncRNA NEAT1	miR-490-3p	VSMCs	AS-specific (ApoE^−/−^ mouse model, high-fat diet induced)	miR-490-3p is an inhibitory target of NEAT1; overexpression of lncRNA NEAT1 promotes VSMCs proliferation and reduces early apoptosis by sponging the miR-490-3p/hnRNPA1 axis	[99]

↓ represent inhibition.

**Table 3:** The role and mechanism of lncRNA in regulating macrophages.

Effect	lncRNA	Targeted miRNA	Model	Context	Molecular Mechanism	References
Macrophage Polarization	lncRNA-M2	---	Macrophages	AS-specific (LPS-induced, M2 differentiation)	STAT3 enhances lncRNA-M2 transcription and histone modification, facilitating M2 differentiation via the PKA/CREB pathway	[106]
	lncRNA-MRGPRF-6:1	---	Macrophages	AS-specific (ox-LDL-induced, M1 polarization)	Regulates macrophage polarization direction through TLR4-MyD88-MAPK pathway; inhibition reduces foam cell formation and M1 polarization	[107]
	lncRNA AFAP1-AS1	---	THP-1 cells	AS-related (LPS/IFN-γ-induced, M1 polarization)	Overexpression promotes M1 polarization and inhibits M2 polarization in LPS/IFN-γ-treated THP-1 cells	[108]
Macrophage Inflammation	lncRNA H19	---	THP-1-derived macrophages	AS-specific (ox-LDL-induced, inflammation/oxidative stress)	Knockdown upregulates ABCA1/PPARα, downregulates NF-κB, slowing inflammation and oxidative stress	[109]
	lncRNA MERRICAL	---	ApoE^−/−^ mice, Ldlr^−/−^ mice, macrophages	AS-specific (high-fat high-sugar diet-induced, macrophage chemotaxis)	Deficiency attenuates CCL3/CCL4 expression, impairs macrophage chemotaxis and inflammatory reactivity	[110]
	lncRNA HOTAIR	---	Macrophages	AS-related (LPS-induced, inflammation/metabolic reprogramming)	Regulates Glut1 expression and glucose uptake via NF-κB, modulates PTEN/HIF1α to connect inflammation and metabolic reprogramming	[111]
Other	lncRNA-MAARS		LDLR^−/−^ mice, macrophages	AS-specific (LDLR^−/−^ mouse model, macrophage apoptosis)	Sequesters HuR in nucleus, modulates p53/caspase-9, silencing attenuates macrophage apoptosis and reduces AS plaque burden	[112]
	lncRNA-SIMALR		Human macrophages	AS-specific (Lipopolysaccharide/IFNγ stimulated, human AS plaque-derived, macrophage apoptosis)	Elevated in atherosclerotic lesions, engages HIF1α to modulate NTN1, suppressing inflammatory macrophage apoptosis	[113]
	lncRNA GAS5	miR-145-5p	ApoE^−/−^ mice Macrophages	AS-specific (high-fat diet-induced, foam cell formation)	Downregulated by phthalates, altering miR-145-5p regulation, increasing lipid uptake and promoting foam cell formation	[114]
	lncRNA AI662270	---	Macrophages	AS-specific (ApoE mice, high-fat diet-induced, cholesterol efflux)	Downregulation amplifies cholesterol efflux, reduces intracellular cholesterol burden, impeding foam cell formation	[115]

**Table 4:** The role and mechanism of lncRNA in regulating lipid metabolism.

Effect	lncRNA	Targeted miRNA	Model	Context	Molecular Mechanism	References
Promotes Lipid Metabolism Disorder	lncRNA-H19	miR-130b	Raw264.7 cells	AS-specific (ox-LDL-induced)	Silencing lncRNA H19 inhibits ox-LDL-induced macrophage adipogenesis and inflammatory response by upregulating miR-130b.	[117]
	lncRNA-H19	miR-146a-5p	RAW264.7 cells	AS-specific (ox-LDL-induced)	Competitively binds miR-146a-5p, relieving its inhibition of ANGPTL4, upregulating ANGPTL4 expression, leading to lipid accumulation and foam cell formation in macrophages	[120]
	lncRNA-HC	---	Hepatocytes	AS-related lipid homeostasis regulation	Forms a complex with hnRNPA2B1, inhibits ABCA1, Cyp7a1, reduces cholesterol excretion	[121]
	lncRNA GM47544	---	ApoE^−/−^ mice	high-cholesterol diet-induced	Regulates ApoC3 expression, affects intracellular triglyceride metabolism; high expression leads to hyperlipidemia and dyslipidemia, promoting arterial plaque development	[122]
Inhibits Lipid Metabolism Disorder	lncRNA-p21	---	ApoE^−/−^ mice, RAW264.7, VSMCs	AS-specific (cholesterol synthesis regulation)	Activates the p53 pathway, inhibits HMG-CoA reductase expression in hepatocytes, reduces endogenous cholesterol generation	[95]
	lncRNA TUG1	---	ApoE^−/−^ mice	AS-specific (atherosclerotic plaque formation context)	Lowers ApoM, ABCA1, ABCG1, thereby elevating TUG1, lowering cholesterol efflux rate, effectively inhibiting AS	[123]
	lncRNA HOXC-AS1	---	THP-1 cells	AS-specific (ox-LDL-induced)	Inhibits ox-LDL-induced cholesterol accumulation	[124]
	lncRNA CDKN2B-AS1	---	THP-1 cells	AS-specific (foam cell formation context)	Inhibits ADAM10 expression in AS to enhance cholesterol efflux, regulates the PPARγ/LXRα signaling pathway to promote ABCA1-mediated cholesterol efflux from macrophages	[125,126]
	lncRNA MeXis	---	RAW264.7 cells, LDLR^−^/^−^, Abca1^flox/flox^ mice	AS-specific (cholesterol efflux regulation, Western diet-induced)	Amplifies LXR-dependent transcriptional activation of ABCA1 expression, synergistically promoting cholesterol efflux	[127]

**Table 5:** The Role and Mechanism of TCM modulates AS by Regulating LncRNA.

Type		lncRNA	Model	Molecular Mechanism	References
Endothelial Cells	Salvianolic acid A	lncRNA NR2F2-AS1	ApoE^−/−^ mice	Inhibits the lncRNA NR2F2-AS1-KDELR axis in VECs, reduces GRP78 secretion, thereby alleviating inflammation-mediated AS	[15]
	Tanshinol	lncRNA TUG1	ApoE^−/−^ mice	Reduces lncRNA TUG1 and increases miR-26a to inhibit endothelial cell apoptosis and reduce AS lesions	[16]
	Curcumin	lncRNA MIAT	HUVECs	Alleviates inflammation in AS mouse models and ox-LDL-induced cell models, and affects cell apoptosis/proliferation by regulating lncRNA MIAT’s epigenetic suppression of miR-124, thereby improving AS	[17]
	Icariin	lncRNA H19	ApoE^−/−^ mice, HUVECs	Mediates the lncRNA H19/miR-148b-3p/ELF5 signaling pathway, inhibits EndMT in ApoE^−/−^ mice and ox-LDL-induced HUVECs, exerting a protective effect against AS	[128]
	Xueguan Ruanhua Wan (Vessel Softening Pill)	lncRNA- TUG1	ApoE^−/−^ mice	Associated with inhibiting p38MAPK signaling pathway activation and downstream T-p38, p-p38 expression, suppressing vascular inflammatory response, regulating blood lipids, inhibiting VECs apoptosis, protecting endothelium	[129]
Vascular Smooth Muscle Cells	Icariin	lncRNA H19	ApoE^−/−^ mice, HA-VSMCs	Upregulates lncRNA H19 expression, acts on PKCβ I, and cascades to downstream molecules Cyclin D1 and MMP-9, ultimately exerting inhibitory effects on HA-VSMCs proliferation and migration	[14]
	Baicalin	lncRNA MEG3	HA-VSMCs	Inhibits the expression of cell cycle-related proteins in lncRNA MEG3-knockdown HA-VSMCs; regulation of the MEG3/p53 signaling pathway suppresses proliferation and promotes apoptosis, ultimately contributing to the inhibition of AS progression	[130]
		lncRNA AK021954	VSMCs	lncRNA AK021954 and FGF18 are involved in the process of Baicalin inhibiting VSMCs proliferation, but the exact molecular mechanism remains unclear	[131]
	Astragaloside IV	lncRNA H19	ApoE^−/−^ mice	Inhibits VSMCs autophagy and mineralization in AS; the mechanism may be related to lncRNA H19 and DUSP5-mediated ERK signaling	[132]
	Asparagus Extract	lncRNA NEAT1	VSMCs	Associated with downregulation of lncRNA NEAT1 and Bmal1/Clock; Asparagus extract, as a dietary supplement, protects VSMCs from acrolein-induced apoptosis	[133]
Macrophages	Tongmai Zhuke Decoction	lncRNA-Cox2	Macrophages	Upregulates lncRNA-Cox2 to inhibit the inflammatory response of macrophages in carotid AS	[18]
	Artemisinin	lncRNA ENSMUST00000099676.4, ENSMUST00000143673.1, ENSMUST00000070085.5, ENSMUST00000224554	ApoE^−/−^ mice	Effectively alleviates AS by regulating the AMPK/mTOR/ULK1 pathway to inhibit macrophage polarization	[134]
	Resveratrol	lncRNA MALAT1	ApoE^−/−^ mice	Exerts anti-AS effects by regulating lncRNA MALAT1, thereby modulating the expression of inflammatory factors IL-6, IL-1β, TNF-α	[135]
	Sinapic Acid	lncRNA-MALAT1	Diabetic AS rats	May alleviate macrophage pyroptosis by downregulating lncRNA-MALAT1 in diabetic AS rats	[136]
Lipid Metabolism	Gypenosides	lncRNA TUG1	ApoE^−/−^ AS mice	Gypenosides may improve hepatic lipid deposition in ApoE^−/−^ AS mice by influencing the lncRNA TUG1/miR-26a axis to interfere with the mitochondrial apoptosis pathway, thereby preventing/treating AS	[137]
	EGCG	lncRNA NONHSAT102202	HepG2 cells	EGCG directly affects key genes in cholesterol balance regulation (LDLR, HMGCR, ACAT2), while upregulating or downregulating numerous lncRNAs, thereby alleviating AS	[138]
	Xiangsha Liujunzi Tang	lnc-HC	ApoE^−/−^ mice	Affects lipid metabolism and prevents AS by influencing lnc-HC to regulate PPARγ-mediated cholesterol metabolism processes	[139]
	Resveratrol	lncRNA-HOTAIR	3T3-L1 preadipocytes	Inhibits lipid droplet formation and accumulation via the AdipoQ-AdipoR1-AMPKα and AdipoQ-AdipoR2-PPARα signaling pathways	[140]
	Fucoxanthin	lncRNA HOXA-AS3, HULC, IPW, PCAT-29, PCAT-43, CA-8	ADSCs	Genetic regulation targeting lncRNAs can regulate adipogenesis	[141]
	Yinchenhao Tang (Artemisia Scoparia Decoction)	lncMEG3	MAFLD mice	Intervenes with lncMEG3 to regulate the miR-21-5p/PPARα signaling pathway, thereby promoting fatty acid β-oxidation to improve metabolic-associated fatty liver disease	[142]

## Data Availability

No new data were created or analyzed in this study. Data sharing is not applicable to this article.
